# Types of geographical distribution of leaf beetles (Chrysomelidae) in Central Europe *

**DOI:** 10.3897/zookeys.157.1798

**Published:** 2011-12-21

**Authors:** Michael Schmitt, Thomas Rönn

**Affiliations:** 1 Ernst-Moritz-Arndt-Universität, Allgemeine & Systematische Zoologie, Anklamer Str. 20, D-17489 Greifswald, Germany; 2 Universitätsklinikum Essen, Institut für Physiologische Chemie, Virchowstr. 171, D-45122 Essen, Germany

**Keywords:** Insecta, Coleoptera, Chrysomelidae, zoogeography, grid maps, faunistics, Central Europe

## Abstract

A comparison of the geographical distribution patterns of 647 species of Chrysomelidae in Central Europe revealed 13 types of distribution: (1) widely distributed, (2) southern, (3) southeastern, (4) southwestern, (5) northern, (6) eastern, (7) south east quarter, (8) south west quarter, (9) fragmented, (10) montane, (11) subalpine & alpine, (12) scattered, (13) unusual, and irregular patterns produced by insufficient data. Some of these distributions are trivial (e. g. northern, eastern, etc., alpine) but others are surprising. Some cannot be explained, e. g. the remarkable gaps in the distribution of *Chrysolina limbata* (Fabricius, 1775) and in *Aphthona nonstriata* (Goeze, 1777). Although our 63.000 records are necessarily tentative, we found that the distribution maps from these data reflect in many cases the common knowledge on the occurrence of leaf beetles in specific areas.

## Introduction

Distribution data of organisms are necessary for basic research, as they provide insights into their potential ecological interactions and the colonisation of a given area. Moreover, comparing distribution patterns, morphological and/or physiological traits can inspire hypotheses on how ecological adaptations and phylogenetic constraints become possible. Such data are also a prerequisite for sound decisions in nature conservation and an integral contribution to applied sciences.

In the autumn of 1987, a group of 18 amateur and professional coleopterists working on leaf beetles decided to co-operate in continuing the faunistics project of Adolf Horion (12.07.1888 – 28.05.1977). He had published a series of 12 volumes on the geographic distribution of beetles in Central Europe between 1941 and 1974 but could not complete his project of treating all coleopteran families. As they considered it necessary to compile the available data on the zoogeography of Chrysomelidae, the 18 enthusiasts formed a working group on leaf beetle faunistics (CHRYFAUN) (those whose names and last names are given in Italics left the group in the meantime): Ulf Arnold (Schöneiche, Germany), Wolfgang Bäse (Rheindorf, Germany), Ron Beenen (Nieuwegein, The Netherlands), Bozidar Drovenik (Ljubljana, Slovenia), *Manfred Döberl* (Abensberg, Germany),Dieter Erber (24.02.1933 - 28.02.2004, Giessen, Germany), Frank Fritzlar (Jena, Germany), *Elisabeth Geiser* (Salzburg, Austria),Uwe Heinig (Berlin, Germany), *Horst Kippenberg* (Herzogenaurach, Germany), Michael Langer (Niederwiesa, Germany), *Winrich Mertens* (Freiburg im Breisgau, Germany), *“*Theo” Michael Schmitt (now in Greifswald, Germany), Matthias Schöller (Berlin, Germany), Dieter Siede (now in Retterath, Germany), Walter Steinhausen (München, Germany), and Andrzej Warchałowski (Wroclaw, Poland).

We built a database with entries either based on voucher specimens or on reliable literature data. ‘Reliable’ was defined as records with geographic coordinates down to one minute. There is hardly a consensus among zoogeographers how to circumscribe “Central Europe“ in scientific terms. Horion (1951, p. III) defined Central Europe “sensu stricto” as comprising Germany, Austria and Czechoslovakia. Political borders are definitively irrelevant for the target organisms but indeed they are relevant for human researchers. In addition, decisions in nature conservation regularly require an evaluation of the rareness and the ecological importance of certain species in a given political area. How rare or special a species in a target area is can only be asserted if distribution data are available for the area of concern and for a wider geographic frame. Thus, we apply a broader concept of Central Europe and focus on a rectangular area comprising 12 countries: Belgium, The Netherlands, Luxembourg, Switzerland, Liechtenstein, Germany, Poland, Austria, Czech Republic, Slovakia, Hungary, and Slovenia. This rectangle lies between 2° and 25°E and 45° and 55°N.

There are numerous ways to visualise geographic distributions, partly due to the fact that there are several possibilities to project the earth surface on a plane map (see, e.g. Snyder 1987). Horion (1965) marked 10° × 10° grid cells with pencil crosses for selected species. Beenen and Winkelman (2002) use a Universal Transverse Mercator (UTM) grid for the Netherlands and plot open and solid circles to indicate records from before and after 1950. In another publication, Beenen et al. (2005) use spots of different diameter for the same purpose. In 1998, Beenen had reported on five different patterns of distribution of Galerucinae in The Netherlands (a: evenly dispersed, b: restricted to sandy soil and limestone, c: restricted to sandy areas with Pleistocene soil and to limestone areas, d: restricted to marshes, e: near the borders of The Netherlands) and presented also maps with an UTM grid. The UTM grid was also used by Silfverberg (1987) in his investigation on the distribution of Chrysomelidae in Finland. Warchałowski (1985) gives the geographical distribution by homogeneous blackening of certain areas of the maps. Gruev and Tomov (2007) plotted individual records on 10 km UTM grid cells for Bulgaria. Besides these maps, also tables of different spatial resolution were used to publish information on geographic distribution of beetles, e. g. by Gruev and Döberl (2005) for the flea beetles (Alticinae) of the Palaearctic subregion, within which they differentiated 13 areas; Köhler and Klausnitzer (1998) for the beetles of Germany in 18 areas; or by Lundberg and Gustafsson (1995) for the beetles of Sweden subdivided in 30 provinces.

We decided to present our results finally as grid maps with fields of size 30‘ east to west and 20‘ north to south. This provides the opportunity to include records of which we do not have precise geographic data but know definitely to which grid cell they belong. Our rectangle contains 1380 cells in total, 1291 include land, at least partly, and 1126 grid cells lie at least partly over a focus country. The grid cells differ somewhat in size. Their N-S extension is 37.12 km, but the length of their E-W axis and consequently their surface area varies, e.g. between 31.80 km (= 1180.42 km²) at 55°N, 35.77 km (= 1327.78 km²) at 50°N, or 39.21 km (= 1456.59 km²) at 45°N.

The members of the working group chose the larger grid cell size as compared to that used in Great Britain (10 × 10 km, Cox 1992) because it allows for the inclusion of more records which could not be georeferenced but only assigned to a grid cell. An approach similar to ours is followed by the Bruchidae/Chrysomelidae Recording Scheme in Great Britain (Cox 1992, 2007). However, their grid cells are 10 km-squares, which means that the spatial resolution is approximately twelve times higher than ours. On the other hand, the area we treat is about five times larger than the UK. This might in part compensate for the coarse resolution in CHRYFAUN.

There are 787 species of Chrysomelidae (s. l., i. e. including 66 bruchids) in checklists, but we have data for only 647 species in 63,136 records for 737 grid cells (57 % of 1291, or 65 % of 1126). Here, we present a progress report on the project “faunistics of Central European seed and leaf beetles”. We hope to show that already at the present state some scientifically interesting results have been attained.

## Material and methods

Records were taken into the CHRYFAUN database from (1) the notes of Adolf Horion, forwarded by Dieter Siede, (2) the collections of Zoologisches Forschungsmuseum Alexander Koenig – ZFMK, Bonn (Germany) and Zoologisches Institut und Museum of Ernst-Moritz-Arndt-Universität – EMAU, Greifswald (Germany), and (3) the private collections of Ron Beenen, Manfred Döberl, Uwe Heinig, Horst Kippenberg, “Theo” Michael Schmitt, Matthias Schoeller, Dieter Siede (see above for locations), and Klaus Renner (Bielefeld, Germany). Literature data were taken from the reports published in Fragmenta faunistica 1932-1998 (Adamczewski, Bartoszynski, Bartowska, Bielawski, Brischke, Burakowski, Ciszkiewicz, Enderlein, Glazek, Goljan, Kapuscinski, Karpinski, Karpowicz, Kinel, Krzeminski, Kulczinski, Nunberg, Maczynski, Makolski, Markowski, Mazurowa, Mroczkowski, Ogloblina, Pawlowski, Pisarski, Podoski, Popek, Raabe, Stobiecki, Szymczakowski, Tenenbaum, Wasowska, Wegrzecki, Wierzbicki), Mitteilungen der Arbeitsgemeinschaft rheinischer Koleopterologen (Baumann, Böhme, Brenner, Eisinger, Franzen, Höhner, Junker, Katschak, Koch, Köhler, Matern, Müller, Siede, Stüben, Stumpf, Wagner, Wenzel, Wunderle), Mitteilungen des Entomologischen Vereins Stuttgart (Bense, Braun, Bretzendorfer, Büche, Dynort, Frank, Gladitsch, Hemmann, Kless, Knapp, Konzelmann, Kostenbader, Krell, Lange, Malzacher, Maus, Reibnitz, Rheinheimer, Roppel, Ulbrich, Weber, Wolf-Schwenninger, Ziegler), Geiser (2001), Gürlich (1992), Gürlich et al. (1995), and Vig (1996). Since our data will be accessible through GBIF-D in the near future, we do not list the above sources in detail. They can be seen on each individual entry of a record in the database.

“Record” means a single collection act for a species, as documented on the label(s) on the pin(s) of the voucher specimen(s), or the equivalent information in a publication. We used only such data which allowed for relating a record to a certain grid cell, and which offered a time specification of “before 1900”, “between 1899 and 1950”, and “after 1949”, or more exact. If there were several specimens on a single pin or a series of several specimens with exactly the same label data, we opened only one “record” and entered the number of specimens in a “remarks” field. The geographical coordinates of the localities were entered exact to the minute when possible. Where we could only assign a locality to a certain grid cell of 20’ × 30’, we used the centre of the grid cell as a dummy in generating distribution maps. In such a record, the assignment of coordinates to the locality was labelled “artificial” in the database.

Our database CHRYFAUN was developed by the first author and was housed at ZFMK until 2009. Since then, the master database is ministered by the first author at EMAU, copies are distributed among the members of the working group. The database software CHRYFAUN is programmed by Hicosoft (Joachim Hilgers, Düsseldorf, Germany) on a MS Visual FoxPro® platform. Distribution maps are produced using DMAP® (by Alan Morton, Penrhynoch, Aberystwyth, Ceredigion, UK).

In the maps produced, grid cells in light yellow indicate those for which we have data. Consequently, we can only speculate on blank areas. As even most common species are not necessarily reported for all covered grid cells, we plot the distribution of the species of interest using red diamonds against the sampled records of all species of the same genus (or a genus with similarly looking species, in case of monotypic genera). The rationale behind this procedure is that collectors would hardly look for a single species and discard specimens of the remaining species of the same genus. Also, we hope to avoid confusing occurrence gaps with report gaps.

Maps were generated for all of the 647 species under study. Of these, 115 were discarded because they were based on less than 10 records. The other maps were compared by eye according to superficial similarity. The maps could be grouped to certain easily circumscribable types, and there were only few intermediates. Afterwards, the types were described as detailed and objective as possible. For this purpose, also the frequency maps of all species were considered. This allowed us to assign each species unequivocally to one of the distribution types.

## Results

### Frequency of records

**Figure 1.**
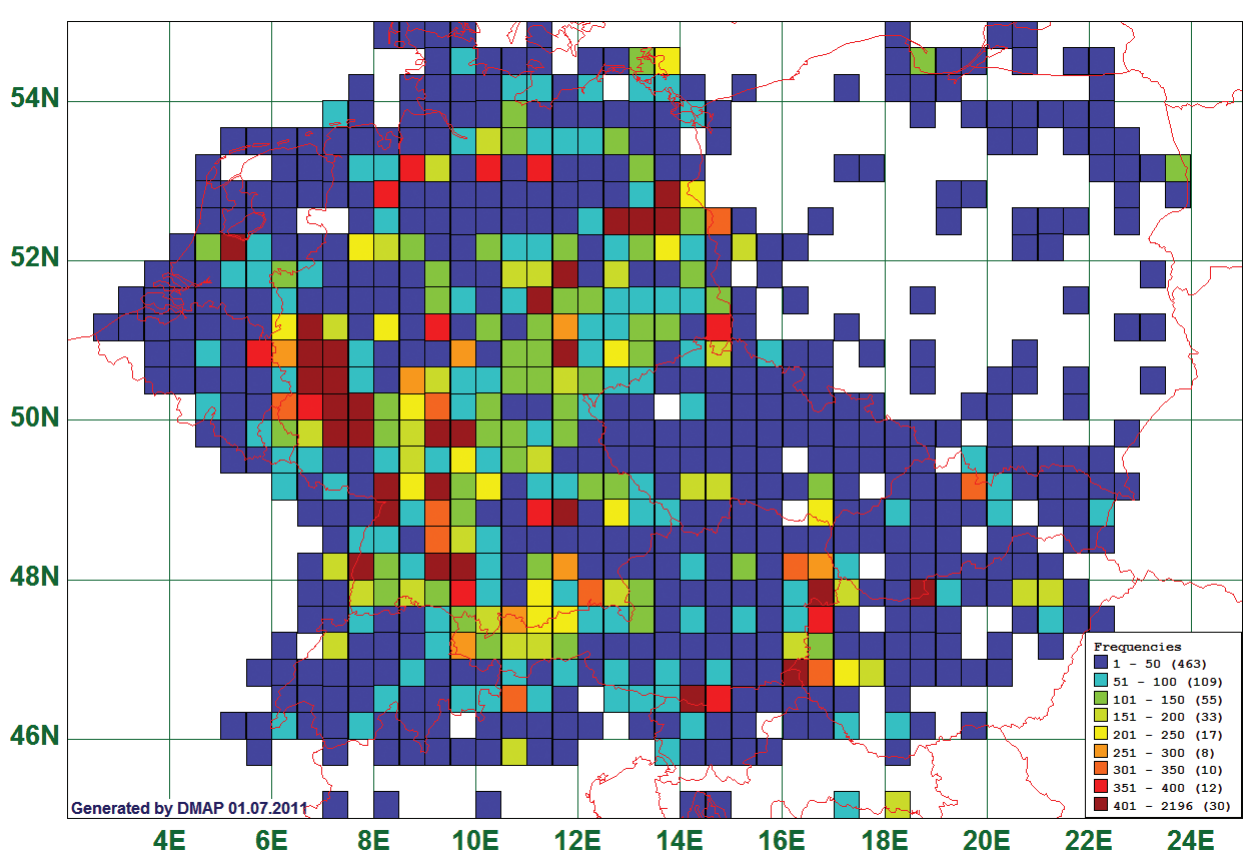
Frequency distribution map, based on 63,136 records of 647 species over 737 grid cells of 20 × 30 geographic minutes. Blank areas mean no records.

The 63,136 records for the 647 target species are not distributed equally over the 737 grid cells of which we have data at all. Fig. 1 shows the frequencies of records plotted on the grid cells. Only for 77 grid cells we have more than 200 records, and only 30 grid cells would allow – cautious - statements for more than 400 species. When scaled differently, it turns out that for 254 grid cells less than 11 records are in our database.

The highest number of records lie in areas (brown squares) where either amateur coleopterists clubs (Rhineland, Baden-Württemberg) or individual collectors (e.g., The Netherlands: Utrecht, Germany: Berlin) are very active, or at touristically and faunistically attractive sites, e. g. Lake Neusiedl in Austria and Hungary.

Species for which we have less than 10 records in the database (114) were only included in the calculations if the records coincide with the zoogeographic information given by (1966), Koch (1992), or Köhler and Klausnitzer (1998). (See Fig. 1)

### Widely distributed: Oulema melanopus (Linnaeus, 1758)

Ninety seven (97) species in our database are reported from all German federal states (Saarland as the smallest state - 2568.7 km² - only facultatively) and additionally from at least four other Central European countries.

Not surprisingly, as an example of a “common” species we present the distribution data of the Cereal Leaf Beetle, *Oulema melanopus* (Linnaeus, 1758), a major crop pest in Central Europe. This species is reported of 209 grid cells, all species of the genus from 315. Records are lacking especially for the Czech Republic and for Slovakia. Very frequently taxonomists did (and still do) not discriminate *Oulema melanopus* from *Oulema duftschmidi* (Redtenbacher, 1874). Therefore, we plot additionally the records reported under this latter name on the map.

**Figure 2.**
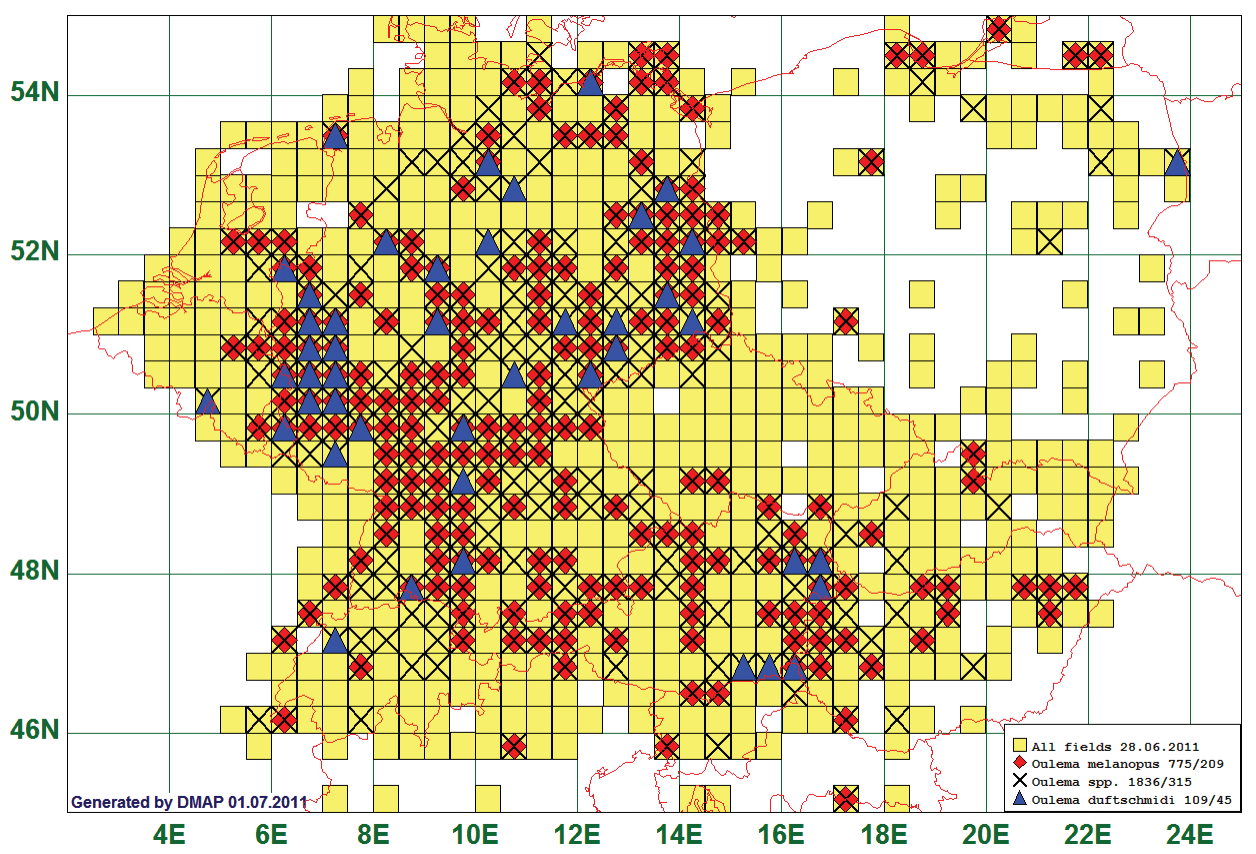
Distribution map of *Oulema melanopus*/*duftschmidi*, based on *775* records for “*Oulema melanopus*”, 109 for “*Oulema duftschmidi*” and 1836 for the genus *Oulema*.

We present this map in spite of the difficult species identification because there is no other species in the database represented by more records. Thus, these records give the clearest picture of a “widespread” species that is deemed to be “common everywhere” in the literature and illustrates the importance of accurate identifications. (See Fig. 2)

### Southern distribution: Altica helianthemi (Allard, 1859)

The distribution of 103 species has a northern border between 50°N and 53°N approximately parallel to the latitude. As an example we present the map of the flea beetle *Altica helianthemi* (Allard, 1859). This species is reported from 34 grid cells, all species of the genus from 255.

**Figure 3.**
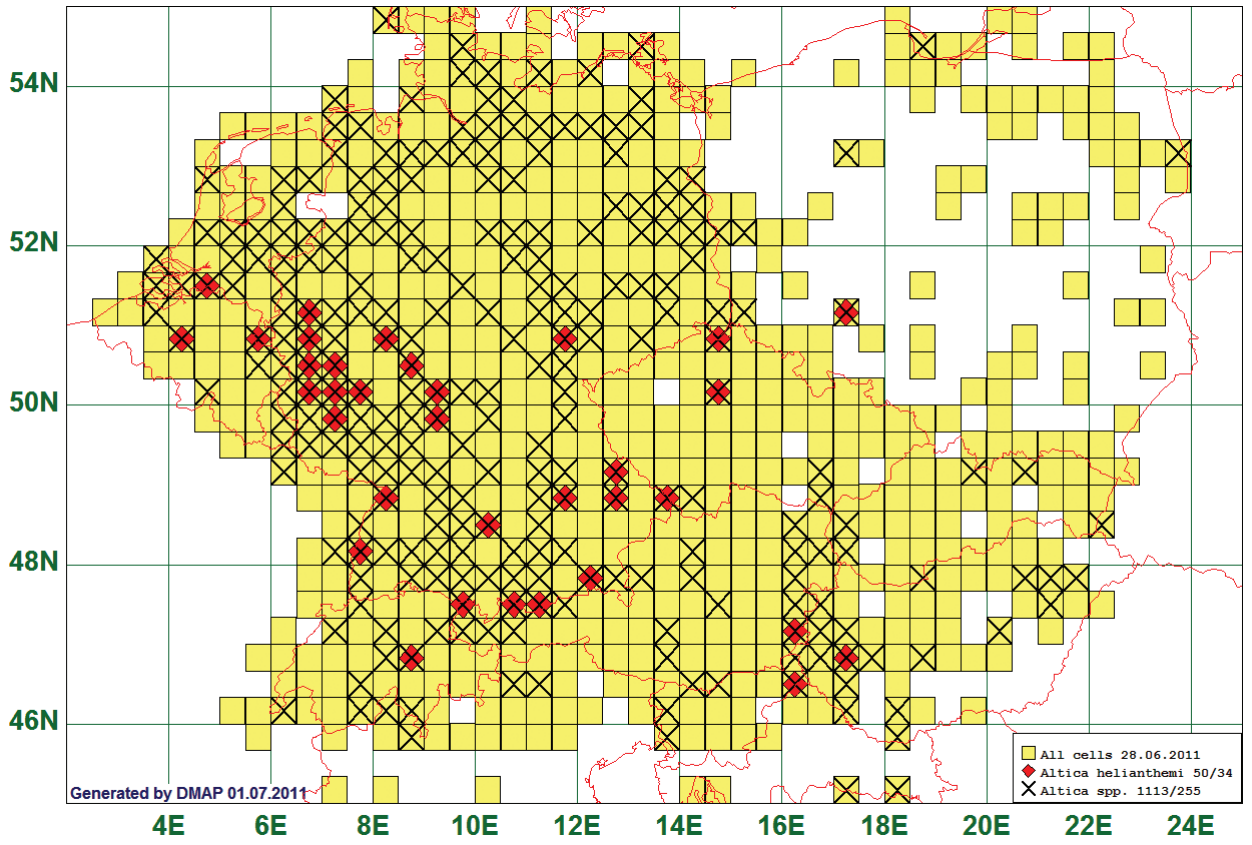
Distribution map of *Altica helianthemi*, based on 50 records for the species and 1113 for the genus *Altica*.

Thirty seven (37) of our 50 records have been either originally identified or later verified by experts on Central European flea beetles (Manfred Döberl, Uwe Heinig, Karl-Heinz Mohr, Dieter Siede). Therefore, we chose this species as an example although it belongs to a group of species in the genus *Altica* which are extraordinarily difficult to discriminate. The records of *Altica helianthemi* fit best to our definition of a “southern” distribution. Most other species have single records lying outside the “southern” domain so that only the overwhelming majority of records show a “southern” pattern. (See Fig. 3)

### South-Eastern distribution: Chrysochus asclepiadeus (Pallas, 1773)

**Figure 4.**
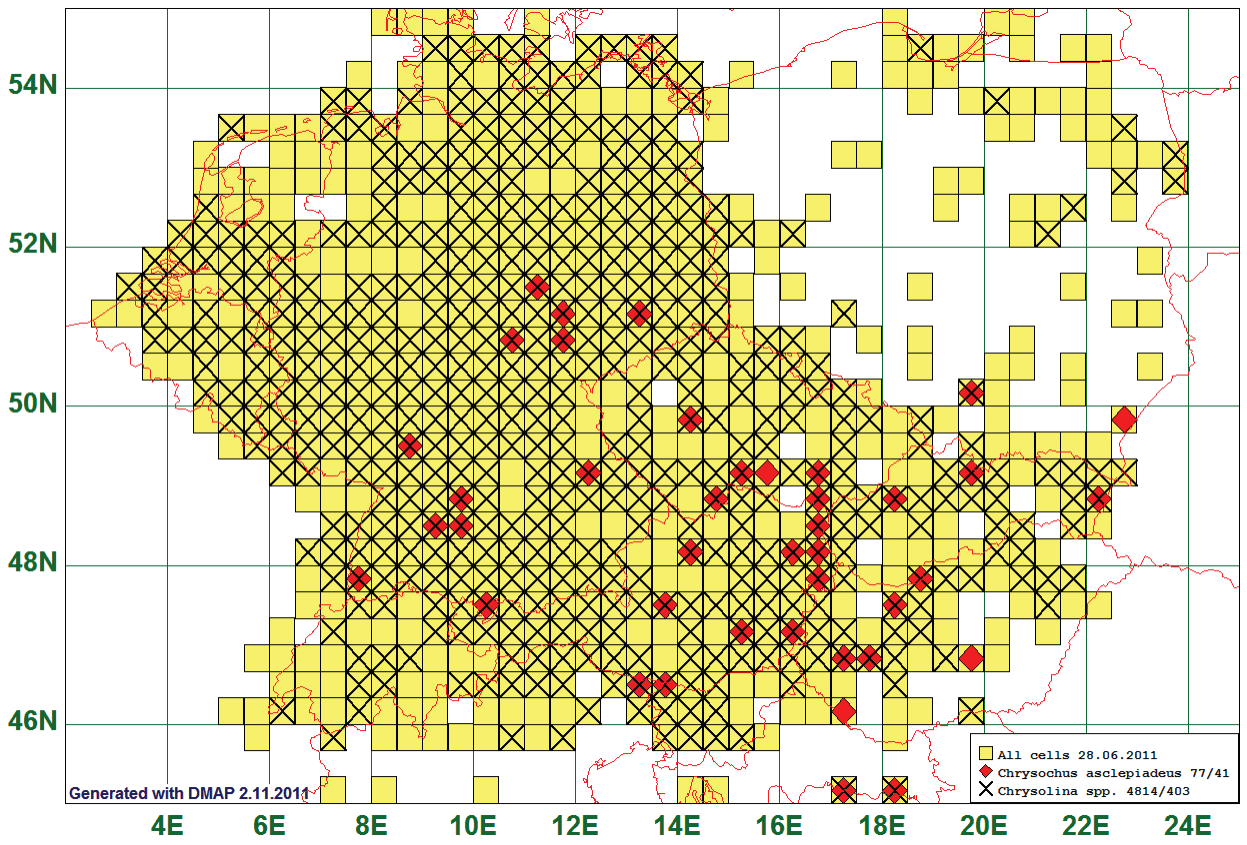
Distribution map of *Chrysochus asclepiadeus*, based on 77 records for the species and 4814 for the genus *Chrysolina*.

Fifty two (52) species had their northern boundary between 51°N and 55°N, stretching from South-West to North-East. As an example we present the map of *Chrysochus asclepiadeus* (Pallas, 1776). This species is reported from 41 grid cells. Since this is the only species of its genus, we plot the records against those of the genus *Chrysolina* (assuming that collectors of *Chrysolina*-species most probably will in the field also take *Chrysochus asclepiadeus*, due to the similar appearance), species of the genus *Chrysolina* are reported from 483 grid cells. (See Fig. 4)

### South-Western Distribution: Timarcha tenebricosa (Fabricius, 1775)

**Figure 5.**
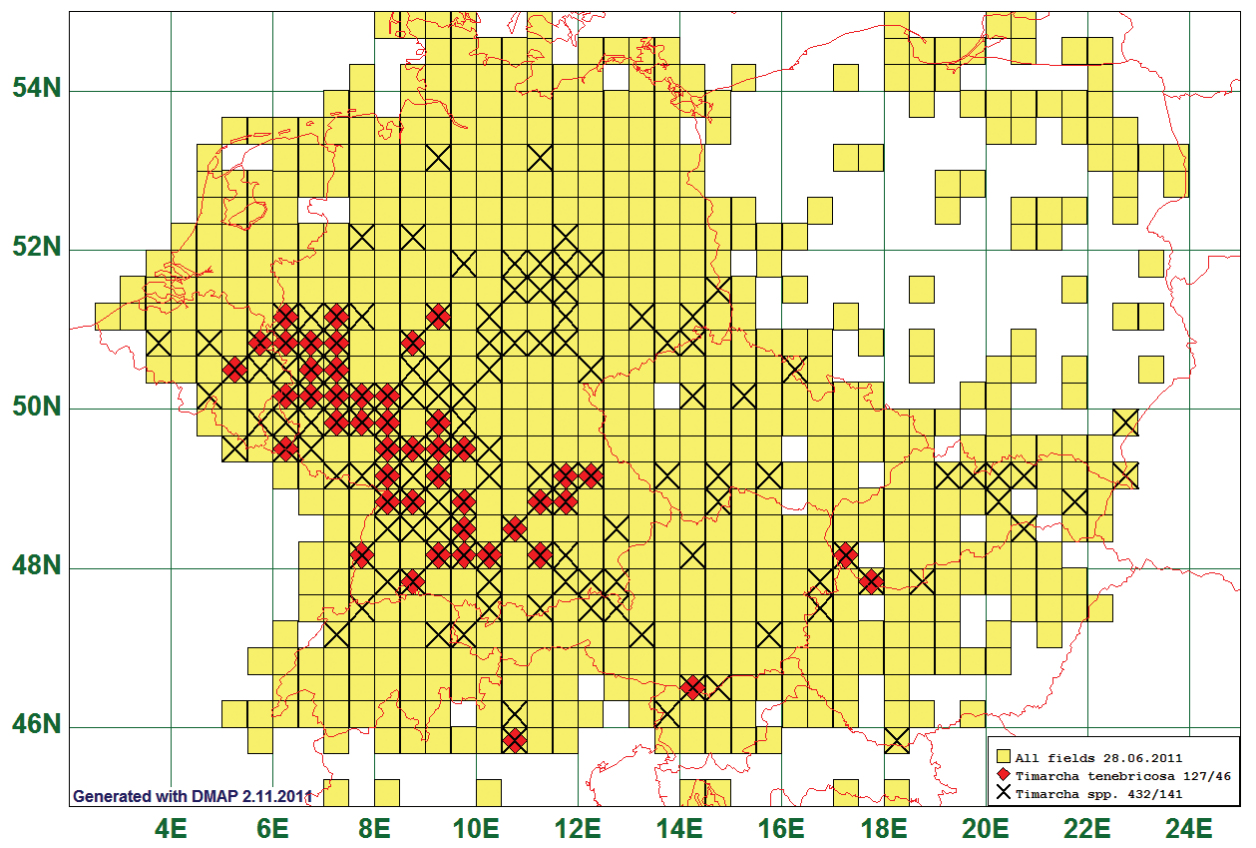
Distribution map of *Timarcha tenebricosa*, based on 127 records for the species and 432 for the genus *Timarcha*.

Twelve species are found only in the south-western part of the study area. Their range extended north between 50° and 54°, while the boundary stretches from North-West to South-East. *Timarcha tenebricosa* is presented as a representative of this type. This species is reported from 46 grid cells, all species of the genus from 141. *Timarcha-tenebricosa*-individuals are the largest leaf beetles in our area. Therefore, we expect that it has not been overlooked so that the pattern of our map shows the real north-eastern boundary of distribution. (See Fig. 5)

### Northern Distribution: Galerucella grisescens (Joannis, 1865)

**Figure 6.**
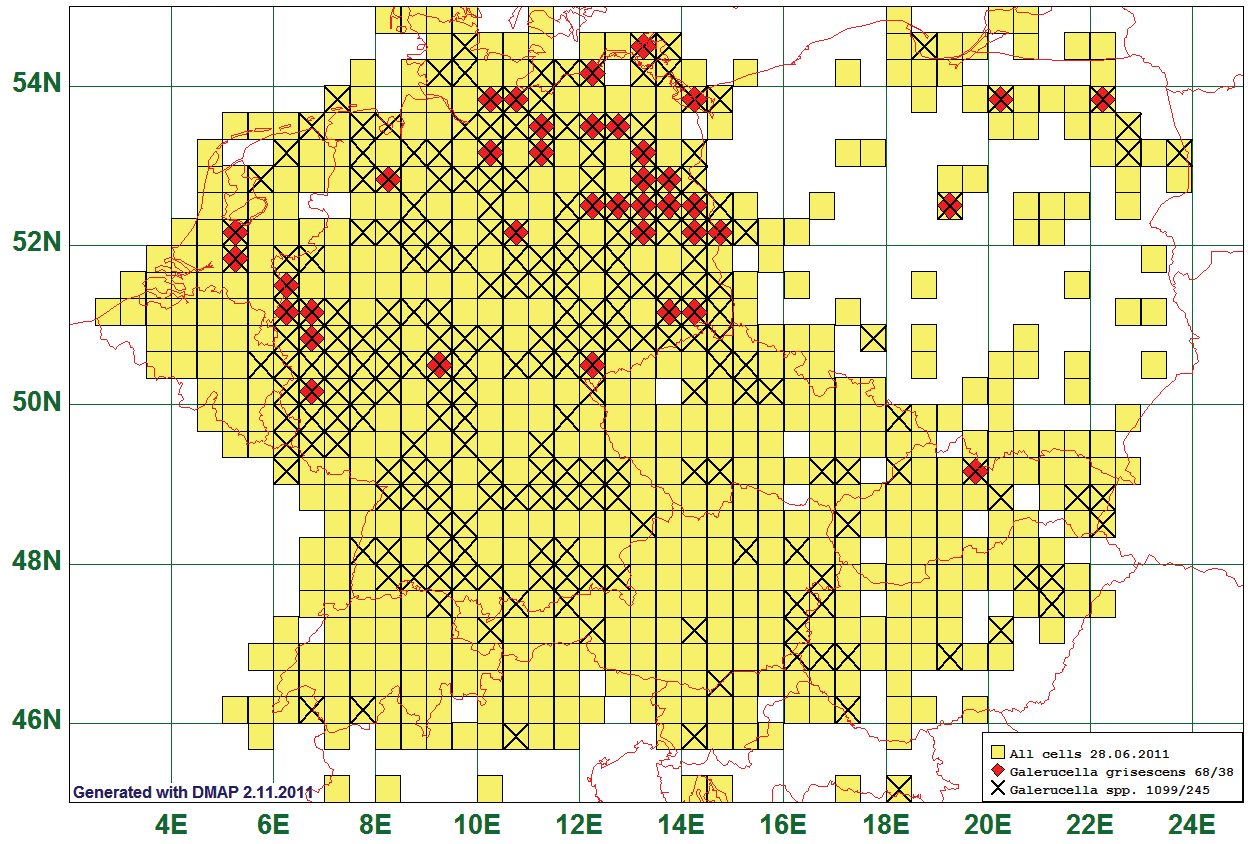
Distribution map of *Galerucella grisescens*, based on 68 records for the species and 1099 for the genus *Galerucella*.

Eight of the listed species are distributed north of 49°N or the abundance of which decreases remarkably between 53°N and 49°N. An example of these species is *Galerucella grisescens* (Joannis, 1865). This species is reported from 38 grid cells, all species of the genus from 245. All 68 records of *Galerucella grisescens* lay north of 49°N, all but one even north of 50°. In the other “northern” species, a certain proportion of records comes from south of 49°N, e.g. 13 of 186 in *Mantura chrysanthemi* (Koch, 1803), or 20 of 103 in *Phyllotreta armoraciae* (Koch, 1803) (most of these southern records lay north of 48° anyway). (See Fig. 6)

### Eastern Distribution: Aphthona nigriscutis Foudras, 1860

**Figure 7.**
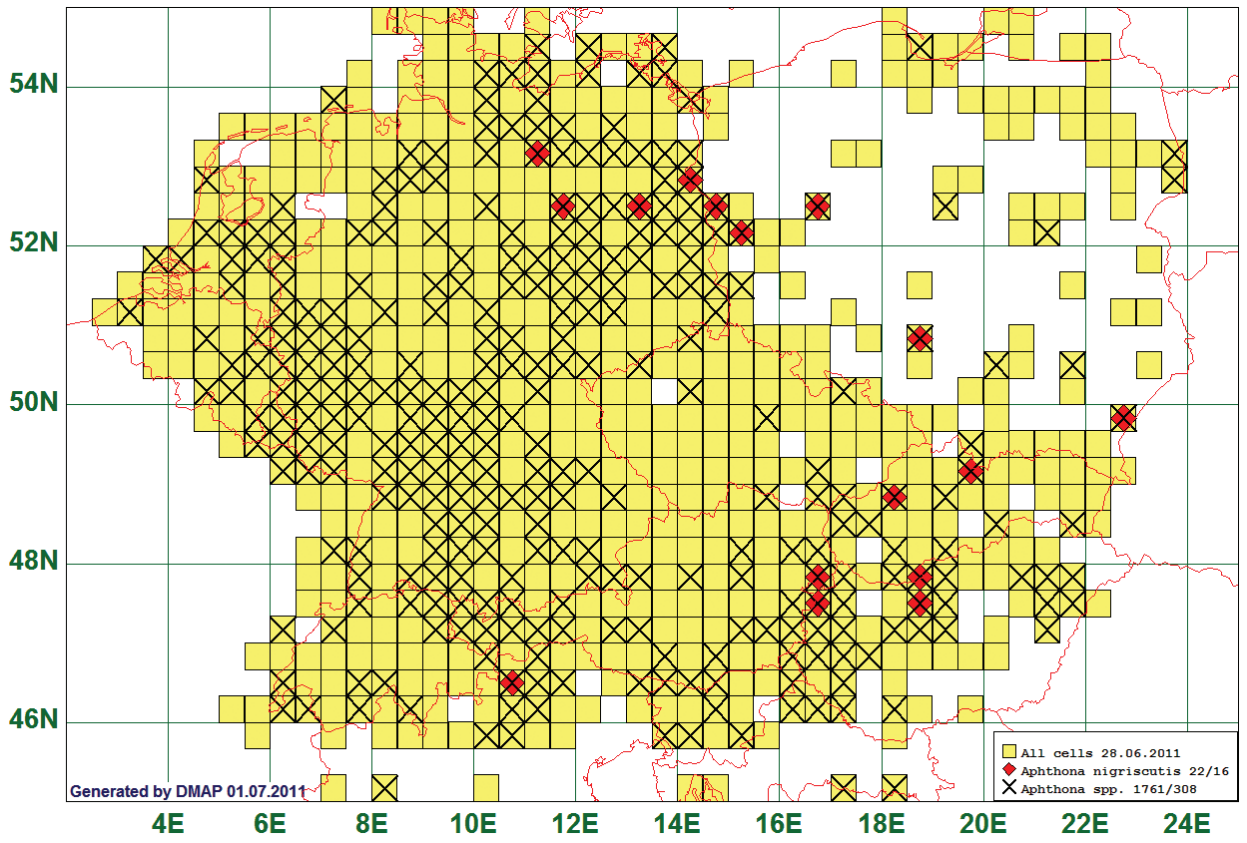
Distribution map of *Aphthona nigriscutis*, based on 22 records for the species and 1761 for the genus *Aphthona*.

Of the 647 species analysed, 16 had a western distribution boundary between 10°E and 14°E. As an example we present the distribution map of *Aphthona nigriscutis* Foudras, 1860. This species is reported from 16 grid cells, all species of the genus from 308. Of the 22 records for this species, 6 lay west of 12°E, and of these, 4 are in grid cell 4256 which covers the Vinschgau in South Tyrol. (See Fig. 7)

### Southeast-quarter Distribution: Crioceris quinquepunctata (Scopoli, 1763)

**Figure 8.**
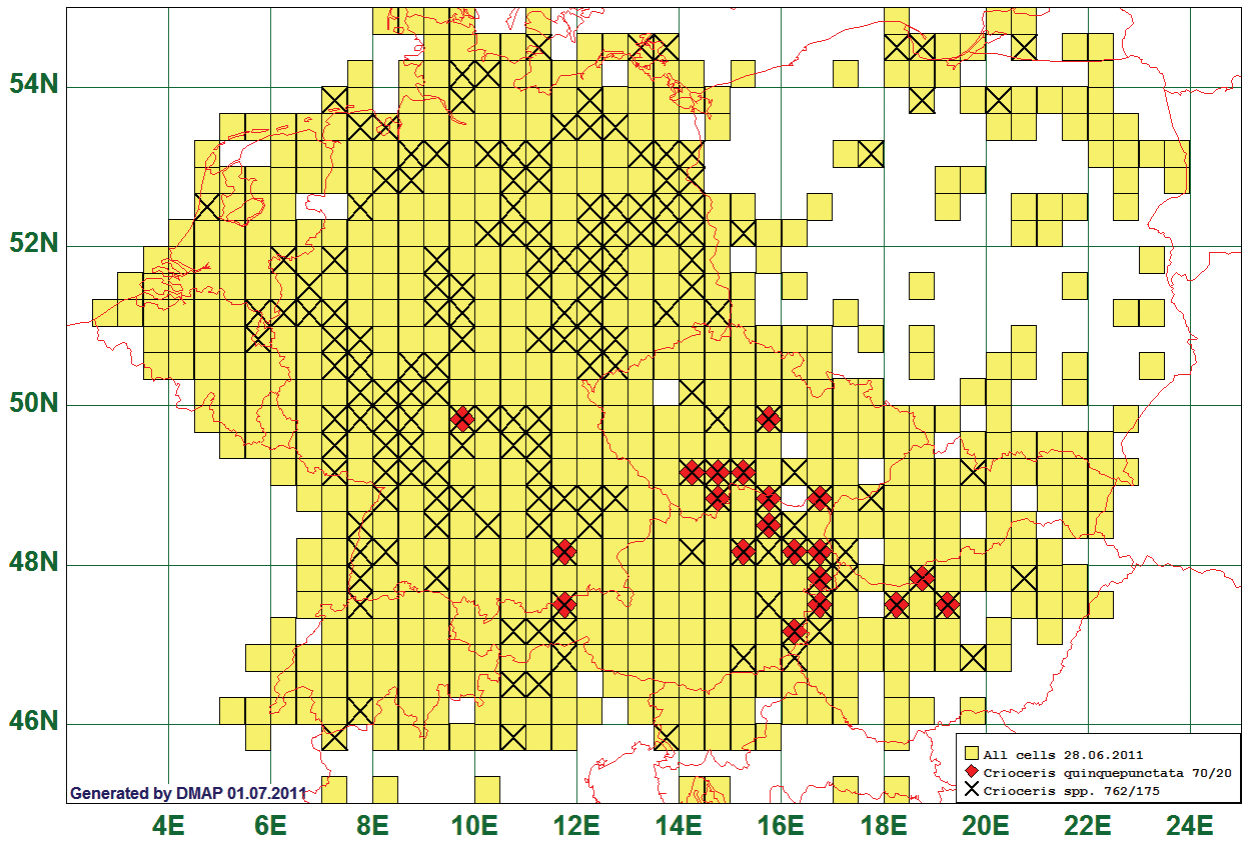
Distribution map of *Crioceris quinquepunctata*, based on 70 records for the species and 762 for the genus *Crioceris*.

52 species occurred only in areas south of 51°N and east of 10°E. The Five-spotted Asparagus Beetle *Crioceris quinquepunctata* (Scopoli, 1763) is given as an example of this type. This species is reported from 20 grid cells, all species of the genus from 175. As the westernmost record represents a single specimen from an *Asparagus*-plantation in Lower Franconia near Würzburg, the natural western boundary of this species lies supposedly more eastern. (See Fig. 8)

### Southwest-quarter Distribution: Bruchidius varius (Olivier, 1795)

**Figure 9.**
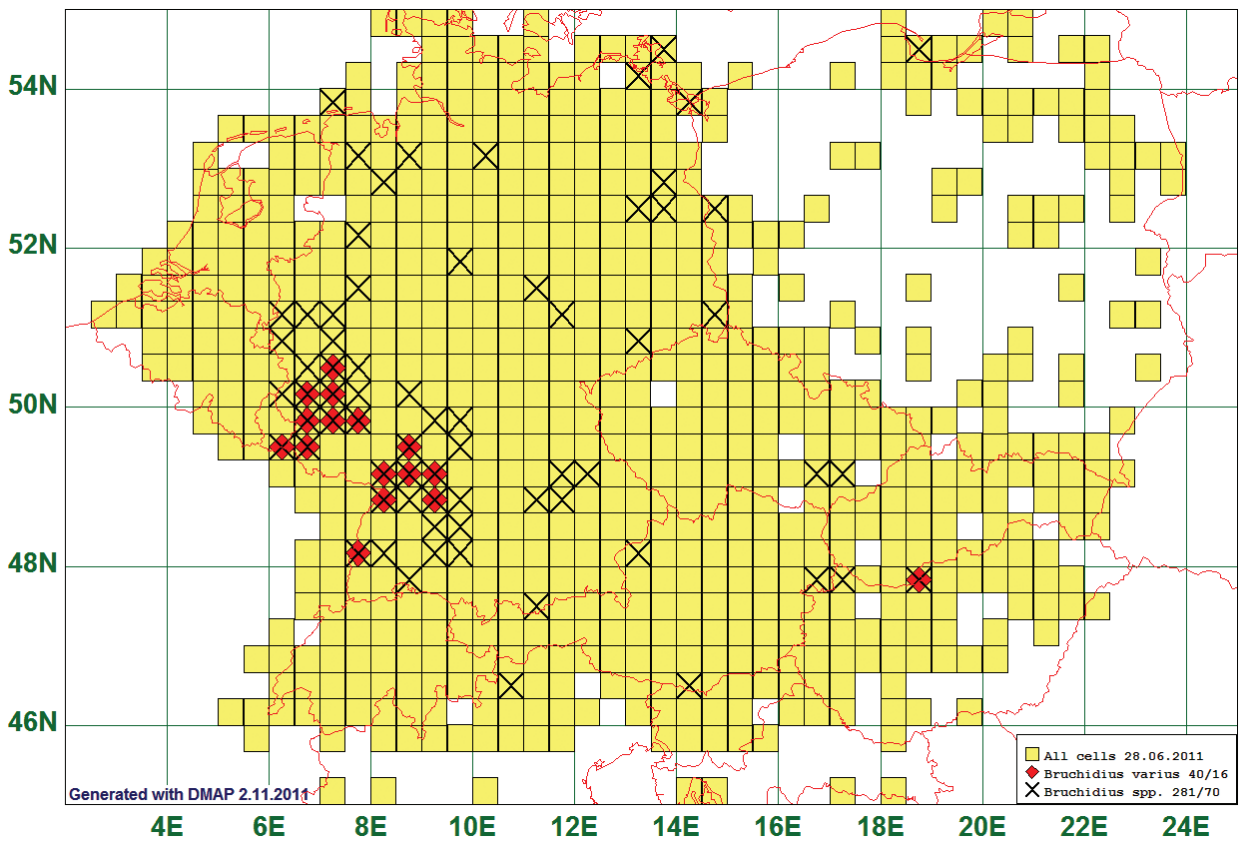
Distribution map of *Bruchidius varius*, based on 40 records for the species and 281 for the genus *Bruchidius*.

Only five species were reported exclusively from areas south of 51°N and west of 10°E. One of them is the seed beetle *Bruchidius varius* (Olivier, 1795) which is given as the example in Fig. 9, it is reported from 16 grid cells, all species of the genus from 70. Possibly this species is not confined to the southwestern area, as indicated by the single record from northern Hungary. (See Fig. 9)

### Fragmented Distribution: Aphthona nonstriata (Goeze, 1777)

**Figure10.**
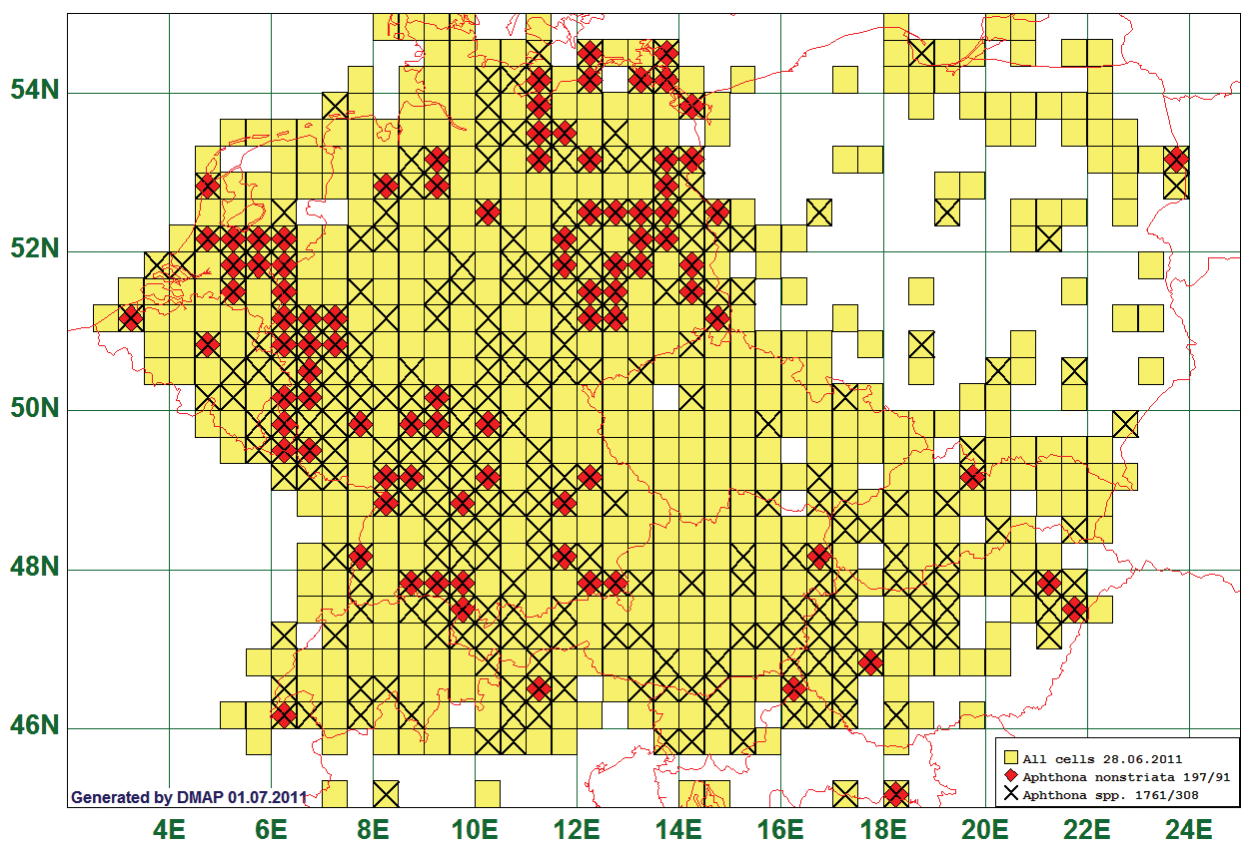
Distribution map of *Aphthona nonstriata*, based on 197 records for the species and 1761 for the genus *Aphthona*.

Of the studied species, 37 showed a surprising distribution pattern. These species are reported from all over Central Europe, but have a remarkable gap, in most cases in Central Germany, Southeast Germany and/or the Alpine region. These gaps cannot plausibly be explained by selective collecting, as congeneric species are reported from these gaps. An example of such a pattern is *Aphthona nonstriata* (Goeze, 1777). This species is reported from 91 grid cells, all species of the genus from 308. The distribution gap in Central Germany is obvious, but also other areas from which congeneric species are reported but no*t A. nonstriata* can be recognised, e. g. in South Germany, in Austria and in Hungary. The example of *Aphthona nonstriata* is especially striking because the gaps cover areas which have been extensively studied by numerous flea beetle specialists. (See Fig. 10)

### Montane Distribution: Oreina alpestris (Schummel, 1844)

**Figure11.**
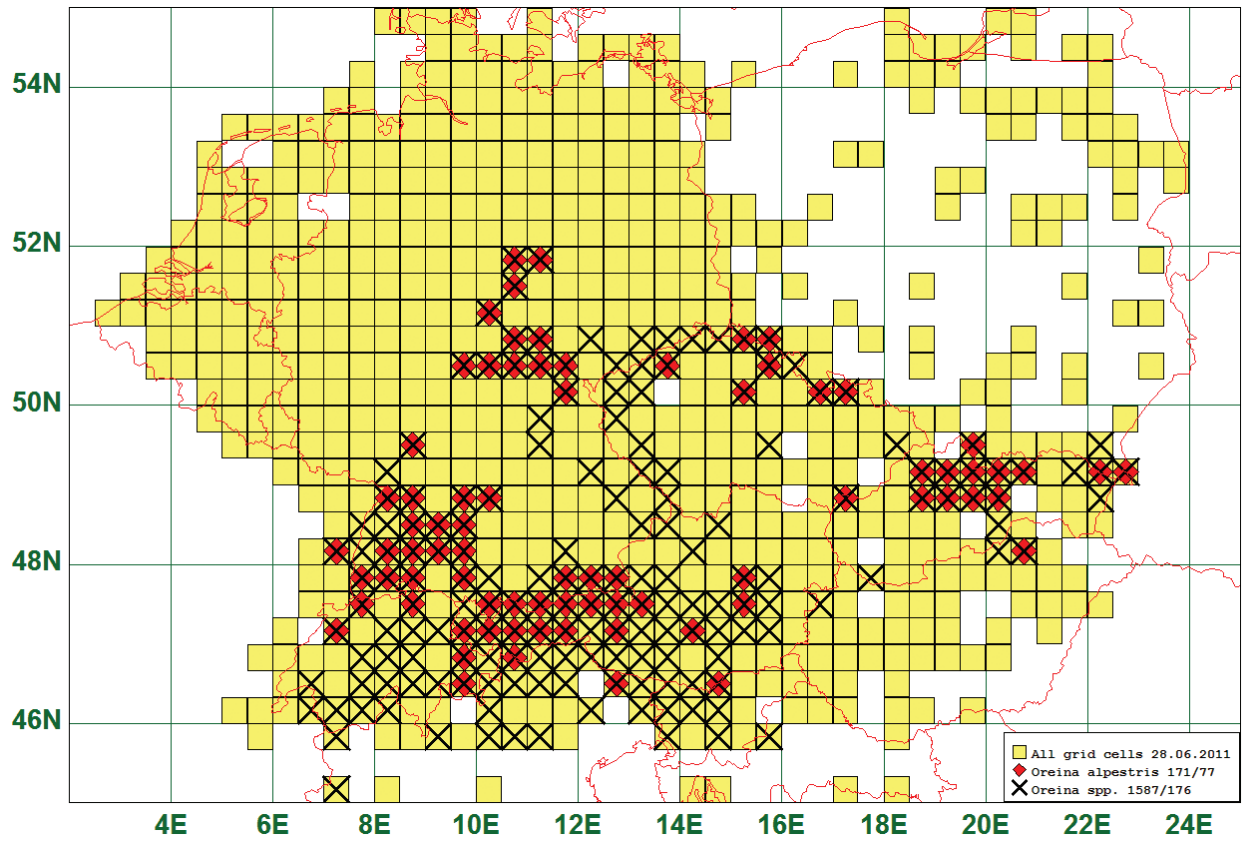
Distribution map of *Oreina alpestris*, based on 171 records for the species and 1587 for the genus *Oreina*.

Eighteen (18) species are distributed in montane areas, i. e. between 200 m and 1500 m a. s. l. As an example we present the distribution map of *Oreina alpestris* (Schummel, 1844) which is found in The Vosges, Black Forest, around the European Alps, in the Harz and the Erz Mountains, and in the Carpathians. This species is reported from 77 grid cells, all species of this genus from 176. It is obvious that all *Oreina*-species are distributed in a similar way. (See Fig. 11)

### Alpine Distribution: Lilioceris tibialis (Villa, 1838)

**Figure 12.**
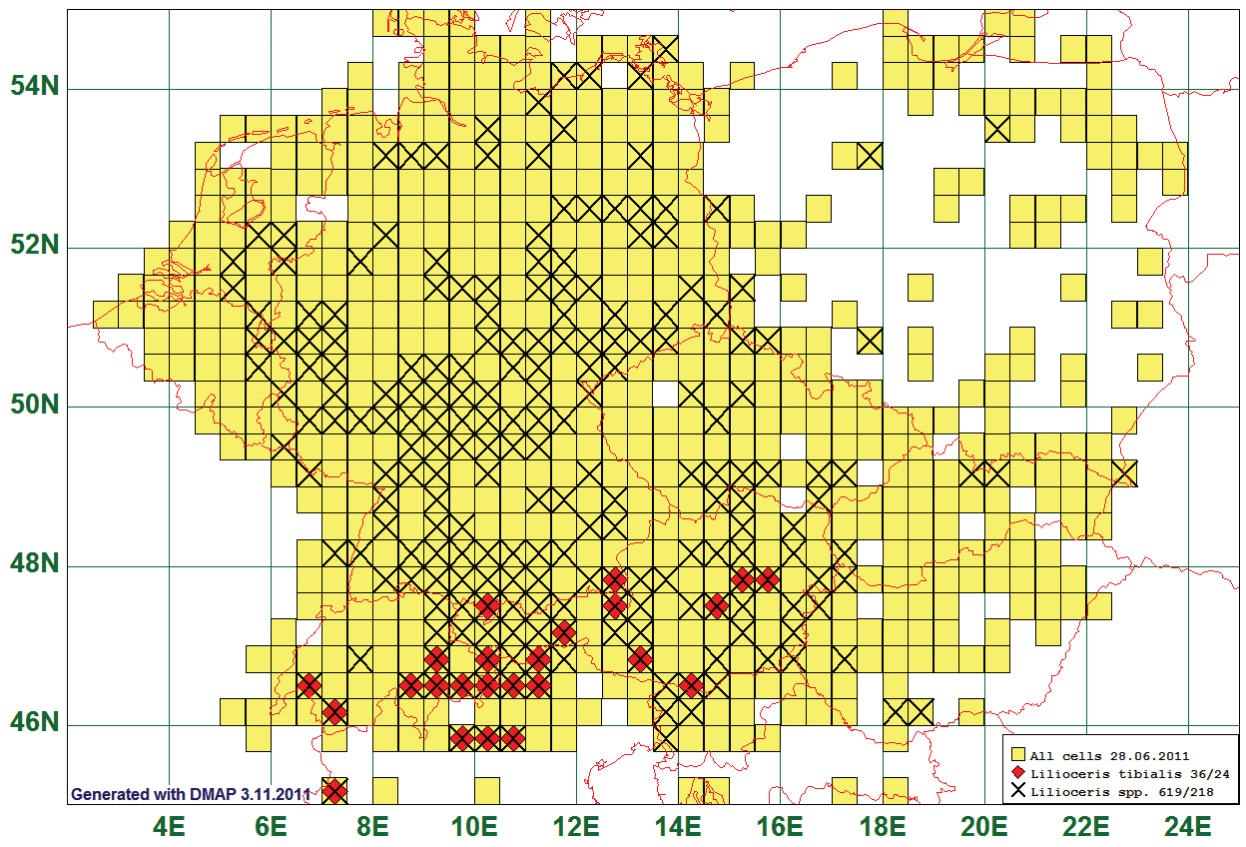
Distribution map of *Lilioceris tibialis*, based on 36 records for the species and 619 for the genus *Lilioceris*.

Distributions restricted to alpine areas, i. e. regions comprising peaks of more than 1500 m a. s. l., were characteristic of 28 species. The example chosen here is *Lilioceris tibialis* (Villa, 1838). This species is reported from 24 grid cells, all species of this genus from 218. Seemingly, the frequencies of *Lilioceris*-spp. decrease towards North, but when generating a frequency map of the 619 records, it turns out that there are grid cells with more than 20 records in the surroundings of Berlin, and even from the eastern part of the island of Rügen there are five findings. (See Fig. 12)

### Scattered Distribution: Chaetocnema aerosa (Letzner, 1846)

**Figure 13.**
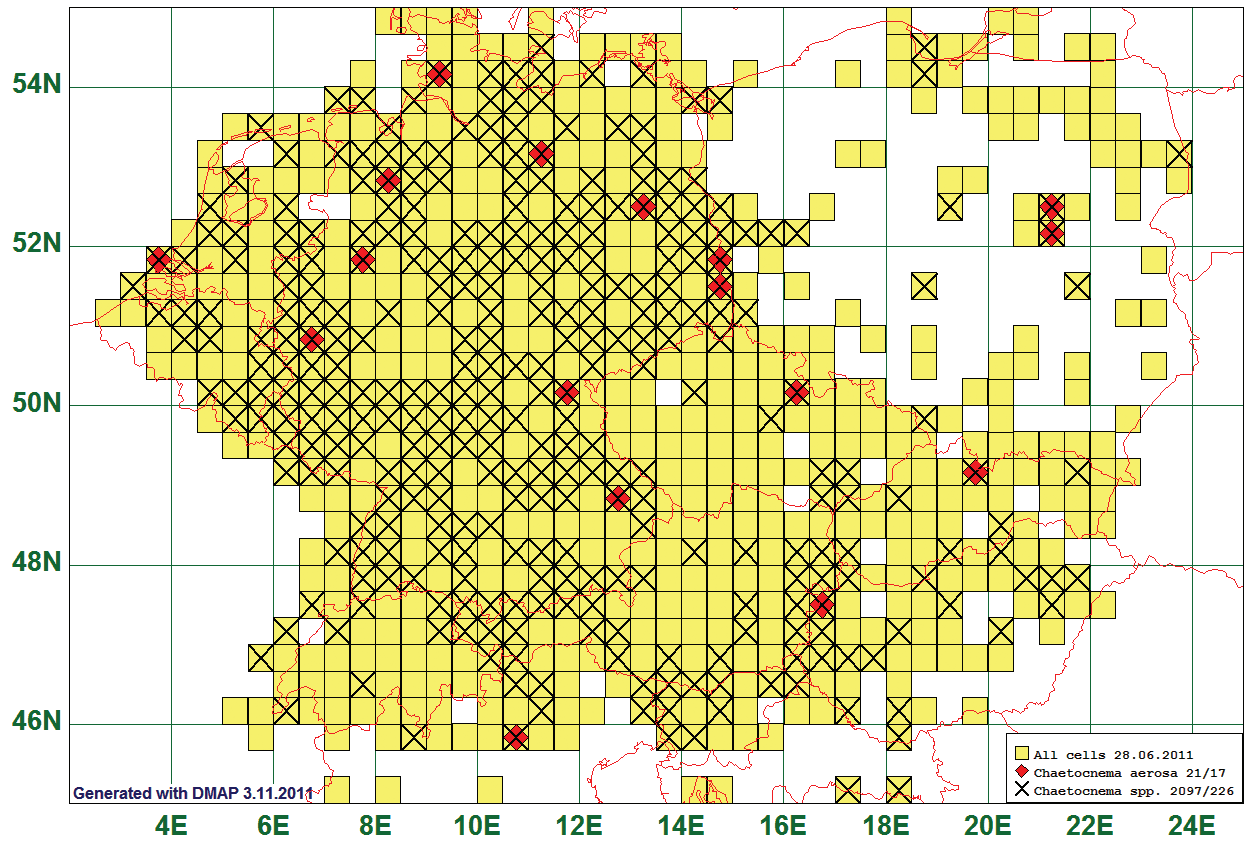
Distribution map of *Chaetocnema aerosa*, based on 21 records for the species and 2097 for all species of the genus.

As “scattered” we define a pattern of few (less than 25) records which are seemingly distributed at random over the map. This is the case in 53 species, *Chaetocnema aerosa* (Letzner, 1846), the example chosen to represent this “pattern”. This species is reported from 17 grid cells, all species of this genus from 226. This distribution pattern is possibly characteristic for a “rare” species, i. e. one with very low abundances. (See Fig. 13)

### Unusual Distribution: Chrysolina limbata (Fabricius, 1775)

**Figure 14.**
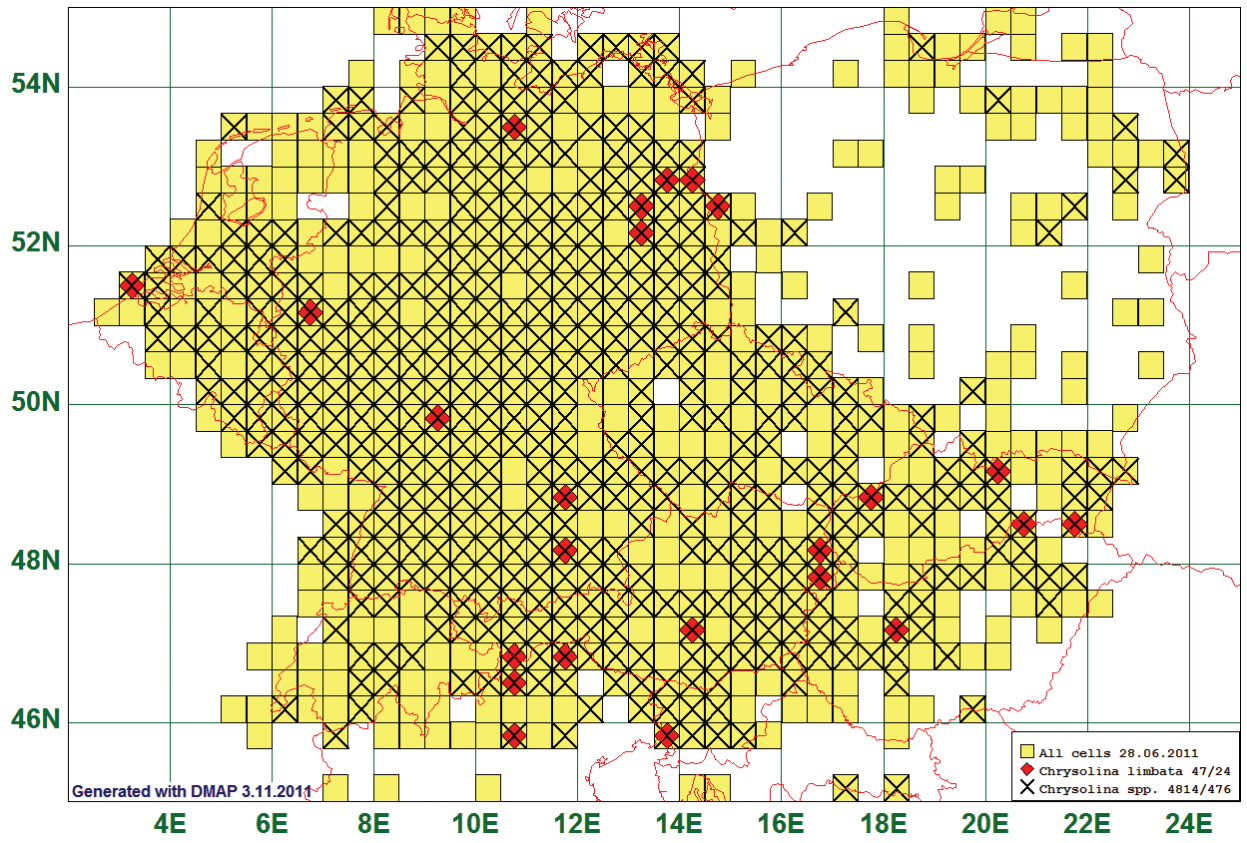
Distribution map of *Chrysolina limbata*, based on 47 records for the species and 4814 records for all species of the genus *Chrysolina*.

Fifty (50) species show a distribution with a marked pattern, which can, however, not plausibly be explained by referring to known patterns. In *Chrysolina limbata* (Fabricius, 1775), some of the marked grid cells are situated around Berlin and in the area of Lake Neusiedl (Austria), the first is residence of several amateur collectors, the second a favoured touristic site, which could explain why beetles of this species were collected right there. But most other records are not correlated with known factors (climate, phytogeography of food plants, collecting activities, orography etc.) pertaining to the probability that a beetle individual gets collected. Similar facts apply for the other 49 cases. *Chrysolina limbata* is reported from 24 grid cells, all species of this genus from 476. (See Fig. 14)

### Proportions of distribution types

**Figure 15.**
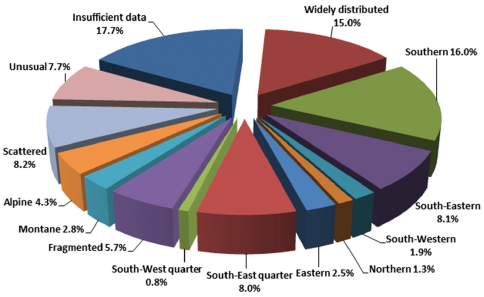
Relative frequency of distribution types of Chrysomelidae in Central Europe.

The types of geographic distributions we distinguish are represented by remarkably different numbers of species, which is shown in Fig. 15.

## Discussion

A first, and unexpected, result of the present study was that the distribution patterns of those 532 species of which we have more than 10 records in our file are not all different or all similar but can easily be grouped into eleven distinct types (widely distributed, southern, south-eastern, south-western, northern, eastern, south-east quarter, south-west quarter, fragmented, montane, and alpine, plus two less distinct forms: irregular and scattered). As it is normal for patterns in nature, there are cases in which the geographical limits are less sharp than the circumscription of the “types” could suggest. But even in these cases, the frequency maps allow for clear assignment of a species pattern to a distribution type, which means that only few records from the edge of a presumed distribution area lie beyond the defining borders.

A second and also remarkable result is that the eleven rather distinct types correspond to zoogeographic patterns described in literature, e.g. in de Lattin (1967). Since we have so few records from Poland and hardly any from France, we refrain from applying de Lattin’s terms which imply a historical zoogeographical interpretation. Our “South-East quarter”-type probably corresponds to de Lattin’s term “pannonian”, our “South-West quarter”-type possibly corresponds to de Lattin’s “atlantomediterranean”, but we find the adoption of these interpretations premature, in spite of the suggestive resemblances. The patterns of certain species, however, differ from the descriptions given in regional catalogues as, e.g., Köhler and Klausnitzer (1998) and Mohr (1966).

Although in numerous cases it is tempting to regard our results as true reflections of real distribution patterns, several caveats must be considered:

(1) As our target area comprises 1291 or at least 1126 grid cells, and we aim at judging on the geographical distribution of 647 species (or ideally 787), it is entirely clear that 63.000 records are definitely too few to allow for justified conclusions. A rough estimate shows that for coverage of 100 records per grid cell and species we would need more than 83 million records. Even if we assume that not all species occur in all grid cells and that less than 100 records would be sufficient, more than 10 million records is a sound estimate for a database meeting all our desires.

(2) Collecting activity of private and professional entomologists is strongly influenced – and was so even more in the past – by political borders and (restricted) freedom to travel. This factor can easily lead to erroneous conclusions on beetle distribution in Central Europe.

(3) An unknown but possibly considerable number of specimens in private and museum collections may be incorrectly determined. This applies more to museum collections than to private ones, since museum curators and collection managers are normally not taxonomic experts of all the taxa they are responsible for. Thus, as many museum specimens come from donations of uncertain taxonomic reliability, or from samples from student projects in the field etc., there are countless causes of mis-identification. These could be detected by the taxonomic specialists in our group in a limited number of cases only. In case of a doubtful record voucher specimens were revised when it was possible. Despite to our effort, a certain degree of uncertainty remains.

(4) There is some arbitrariness in the assignment of a “distribution type”. Although we tried to define these types as clear as possible, we had to cope with cases in which all records but one or extremely few fit into one of our types, and in which we decided to ignore these “aberrant” records (as e.g., with *Bruchidius varius*, see Fig. 9). It can, however, well be that a more complete set of records will prove these decisions wrong, as the “aberrant” records might be indications of a wider or differently shaped distribution.

(5) Similar as above, additional records may lead to a distribution type different from the one we assigned. This was already the case when 9656 additional records were entered into the database after one of us (T.R.) had completed his diploma thesis in 2008 (“Historisch-zoogeographische Analyse der rezenten Verbreitung der Blattkäfer (Coleoptera: Chrysomelidae) in Mitteleuropa”, Rheinische Friedrich-Wilhelms-Universität Bonn, Germany). We found 97 instead of 90 species “widely” distributed, 103 instead of 116 “southern”, 52 instead of 51 “south-eastern”, 37 instead of 36 “fragmented”, 18 instead of 16 “montane”, and 53 instead of 52 “scattered”.

(6) Data density varies extremely with respect to species and areas, as can be seen from Fig. 1. Private and museum collections (which are normally composed of several private collections over time) contain specimens according to individual biases and different scientific purposes. One example are the seed beetles which were regarded a separate family, Bruchidae, and consequently ignored by most leaf beetle enthusiasts. Thus, they are notoriously underrepresented in our data. Another aspect is the low motivation of amateur collectors to collect, mount, label and report “common” species. This is the only plausible explanation for the incompleteness of the records for *Oulema melanopus*/*duftschmidi* (see Fig. 2) and other species which we would expect from all grid cells. But even the extremely dense data yielded by the Oxfordshire Biological Recording Scheme for Oxfordshire show coverage of only about 14 % of all the 2 × 2 km grid cells of the map by records for *Oulema melanopus* (Campbell 1980). The 1992 progress report of the “Bruchidae/Chrysomelidae Recording Scheme” for Great Britain was based on 1800 of its 3033 10-km-squares of which only 563 (Cox 1992) or 780 (Cox 2007) listed “*Oulema melanopus*”. This shows that also there a “common” and “widespread” species is by far not recorded from all areas where it is supposedly present.

### Remark on taxonomy and nomenclature of Oulema melanopus/duftschmidi/rufocyanea.

Berti (1989) published her decision to split the traditionally accepted species *Oulema melanopus* (Linnaeus, 1758) into two, based on her investigation of more than 570 specimens labelled *Oulema melanopus* of the collection of the Muséum National d'Histoire Naturelle (MNHN) at Paris. As she found that the oldest available name for the “new” species is *Oulema duftschmidi* (Redtenbacher, 1874) and no type specimen could be found, she designated a neotype, kept at MNHN. Cox (1995) stated that the “new” species has to bear the name *Oulema rufocyanea* (Suffrian, 1847), since he held the opinion that *Oulema duftschmidi* and *Oulema rufocyanea* are synonyms. An up to now unpublished molecular study (Susanne Dobler, University of Hamburg, pers.comm. 2009) revealed that all tested specimens of *Oulema rufocyanea* were conspecific with *Oulema duftschmidi*, thus corroborating Cox’ statement. Consequently, the correct name of the “new” species should indeed be *Oulema rufocyanea*. Petitpierre (2000) and Warchałowski (2010), however, listed three similar *Oulema*-species for the Fauna Iberica or for the Palaearctic region, respectively, *Oulema duftschmidi*, *Oulema melanopus*, and *Oulema rufocyanea*. This could mean that there is a third species which must not bear the name *Oulema rufocyanea* as long as this name has to be applied to the species named *Oulema duftschmidi* by Berti. As there are three specimens of the *Oulema*-*melanopus*-complex (one from Spain, two from West-Germany) in Dieter Siede’s collection (Retterath, Germany) with male genitalia corresponding to neither alternative depicted in Berti (1989), we leave the question open as to the number of species in the *Oulema*-*melanopus*-complex and which have to be the correct names for them.

For the purpose of the present paper the only relevant aspect of the records labelled “*Oulema melanopus*” is the fact that one would expect to find this “species” in literally all grid cells if our database were as complete as it should be.

Despite these limitations, of which most have been discussed by Geiser (2001b, 2005) already, a great proportion of our results is in line with those found in related scientific literature. However, we must keep in mind that “literature data” arose from collecting activities of individual entomologists and are thus prone to be influenced by the same factors as our data. Other findings, however, may provide more relevant corroborations of our results. The available distribution maps of host plants (Bundesamt für Naturschutz) in Germany are in accordance with the geographical distribution of their guests produced from our data. It turns out that no leaf beetle record is present in a grid cell in Germany in which its food plant is not present. The distribution of certain specialists coincide exactly with the occurrence of their food plant, e. g. the flea beetle *Psylliodes marcida* (Illiger, 1807) and its food plant *Cakile maritima* Scopoli, 1772. Especially interesting is a gap of ca. 300 km in the distribution of *Artemisia campestris* Linnaeus, 1753, the food plant of *Galeruca interrupta* Illiger, 1802. The distribution of records of this beetle species shows exactly such a gap in the same area as the gap in its food plant. It is also worth mentioning that just those species turned out to occur in montane areas or in the alpine regions, respectively, that are characterised as restricted to these areas by countless experienced field entomologists.

The fact that so many species (34.8 %) are distributed in the southern part of our study area (Fig. 15) is congruent with the general decline in species richness from South(-West) to North(-East)and, as, e.g., demonstrated by the zoogeographic data reported in De Lattin (1967, pp. 420ff.) for Rhopalocera in Palatia and other Lepidoptera in the Western Palaearctic. Here, more than 50 % of the species are assigned to a southern (Mediterranean) type of geographical distribution. Silfverberg (1985) mentioned a parallel decline in Finland, which is evident from the 76 grid maps he published in 1987 on species of the subfamilies Donaciinae, Criocerinae, Orsodacninae, Synetinae, Zeugophorinae, Clytrinae and Cryptocephalinae.

The distribution data of most species fit remarkably well to the faunistic literature. For some cases, however, we have no plausible explanation at hand, other cases differ from published statements. Köhler and Klausnitzer (1998) state that *Chrysomela cuprea* Fabricius, 1775 should not occur in the German federal state of Mecklenburg-Vorpommern, from where we have records; or differing from their table we have data for *Psylliodes sophiae* Heikertinger, 1914 also from Bavaria and from Lake Neusiedl. Other than Mohr (1966), we found *Altica brevicollis* Foudras, 1816 also from northern parts of Germany, *Crioceris quatuordecimpunctata* (Scopoli, 1763) also from Schleswig-Holstein and Hessia, *Cryptocephalus nitidus* (Linnaeus, 1758) also in Northern Italy and Slovenia, and *Dibolia depressiuscula* Letzner, 1847 – which is said to occur in whole Central Europe – only south-west of a line from Bonn to Frankfurt an der Oder. Since we do not expect a bias in the selectivity of the collectors of our data especially in these conflicting cases, we are confident that they do not represent “noise” but provide a rewarding basis for future research.

As stated above, more data are needed. However, except for few special cases, no additional collecting in the field is necessary. Our experience in the course of the present study is that private and museum collections harbour enough data to backfill our database up to the intended amount. Thus, we are confident that we can retrieve this buried treasure of knowledge with joint effort.

## Appendix

List of species, assigned to the distribution types.

Species of which we have less than 10 records (116): *Altica ampelophaga* (Guérin-Meneville, 1858), *Altica cornivora* Kral, 1969, *Altica ericeti* (Allard, 1859), *Altica longicollis* (Allard, 1860), *Aphthona aeneomicans* Allard, 1875, *Aphthona beckeri* Jacobson, 1895, *Aphthona czwallinae* Weise, 1888, *Aphthona delicatula* Foudras, 1860, *Aphthona erichsoni* (Zetterstedt, 1838), *Aphthona illigeri* Bedel, 1898, *Argopus bicolor* Fischer, 1824, *Argopus nigritarsis* (Gebler, 1823), *Batophila fallax* Weise, 1888, *Bruchidius bimaculatus* (Olivier, 1795), *Bruchidius dispar* (Gyllenhal, 1833), *Bruchidius lividimanus* (Gyllenhal, 1833), *Bruchidius nanus* (Germar, 1824), *Bruchidius pauper* (Boheman, 1829), *Bruchidius seminaries* (Linnaeus, 1767), *Bruchus ervi* Frölich, 1799, *Bruchus griseomaculatus* Gyllenhal, 1833, *Bruchus lentis* Frölich, 1799, *Bruchus pisorum* (Linnaeus, 1758), *Bruchus sibiricus* Germar, 1824, *Bruchus signaticornis* Gyllenhal, 1833, *Bruchus venustus* Fahraeus, 1839, *Callosobruchus chinensis* (Linnaeus, 1758), *Cassida aurora* Weise, 1907, *Cassida bergeali* Bordy, 1995, *Cassida lineola* Creutzer, 1799, *Cassida seladonia* Gyllenhal, 1827, *Chaetocnema subcoerulea* (Kutschera, 1864), *Chrysolina americana* (Linnaeus, 1758), *Chrysolina fimbrialis* (Kuester, 1845), *Chrysolina globipennis* (Suffrian, 1851), *Chrysolina grossa* (Fabricius, 1792), *Chrysolina morio* (Krynitskii, 1832), *Chrysolina purpurascens* (Germar, 1817), *Chrysolina quadrigemina* (Suffrian, 1851), *Chrysolina reitteri* (Weise, 1884), *Chrysolina schneideri* (Weise, 1882), *Cryptocephalus bohemius* Drapiez, 1819, *Cryptocephalus carinthiacus* Suffrian, 1848, *Cryptocephalus gamma* Herrich-Schaeffer, 1829, *Cryptocephalus loreyi* Solier, 1836, *Cryptocephalus macellus* Suffrian, 1860, *Cryptocephalus marginellus* Olivier, 1791, *Cryptocephalus octacosmus* Bedel, 1891, *Cryptocephalus octomaculatus* Rossi, 1790, *Cryptocephalus quadripunctatus* Olivier, 1808, *Cryptocephalus trimaculatus* Rossi, 1790, *Cryptocephalus turcicus* Suffrian, 1847, *Cryptocephalus villosulus* Suffrian, 1847, *Dibolia alpestris* Mohr, 1981, *Donacia brevitarsis* Thomson, 1884, *Donacia reticulata* Gyllenhal, 1817, *Euluperus xanthopus* (Duftschmid, 1825), *Galerucella sagittariae* (Gyllenhal, 1813), *Gonioctena flavicornis* (Suffrian, 1851), *Gonioctena kaufmanni* (Miller, 1880), *Gonioctena variabilis* (Olivier, 1790), *Hermaeophaga cicatrix* (Illiger, 1807), *Hydrothassa flavocincta* (Brullé, 1832), *Lilioceris schneideri* (Weise, 1900), *Longitarsus aeneicollis* (Faldermann, 1837), *Longitarsus albineus* (Foudras, 1859), *Longitarsus bertii* Leonardi, 1973, *Longitarsus callidus* Warchałowski, 1967, *Longitarsus celticus* Leonardi, 1975, *Longitarsus fuscoaeneus* Redtenbacher, 1849, *Longitarsus longipennis* Kutschera, 1863, *Longitarsus nigrocillus* Motschulsky, 1849, *Longitarsus obliteratus* (Rosenhauer, 1847), *Longitarsus pallidicornis* Kutschera, 1863, *Longitarsus pinguis* Weise, 1888, *Longitarsus rectilineatus* (Foudras, 1860), *Longitarsus strigicollis* Wollaston, 1864, *Longitarsus substriatus* Kutschera, 1863, *Longitarsus tristis* Weise, 1888, *Longitarsus weisei* Guillebeau, 1895, *Luperus carniolicus* Kiesenwetter, 1861, *Longitarsus flaviceps* Apfelbeck, 1912, *Longitarsus nigripes* Kiesenwetter, 1861, *Mantura ambigua* (Kutschera, 1862), *Minota alpina* Biondi, 1986, *Minota carpathica* Heikertinger, 1911, *Minota halmae* (Apfelbeck, 1906), *Minota impuncticollis* (Allard, 1860), *Neocrepidodera basalis* (Daniel, 1900), *Neocrepidodera brevicollis* (Daniel, 1904), *Neocrepidodera crassicornis* (Faldermann, 1837), *Neocrepidodera impressa* (Fabricius, 1801), *Neocrepidodera interpunctata* (Motschulsky, 1859), *Neocrepidodera simplicipes* (Kutschera, 1860), *Oreina caerulea* (Olivier, 1807), *Oreina elongata* (Suffrian, 1851), *Oreina plagiata* (Suffrian, 1861), *Oreina tristis* (Fabricius, 1792), *Orestia aubei* Allard, 1859, *Orestia electra* Gredler, 1868, *Oulema septentrionis* (Weise, 1880), *Pachybrachis carpathicus* Rey, 1883, *Pachybrachis pallidulus* Suffrian, 1851, *Phratora polaris* (Schneider, 1886), *Phyllotreta acutecarinata* Heikertinger, 1941, *Pachybrachis consobrina* (Curtis, 1837), *Pachybrachis hochetlingeri* Fleischer, 1917, *Pachybrachis nigripes* (Fabricius, 1775), *Pachybrachis variipennis* (Boieldieu, 1859), *Pachybrachis ziegleri* Lohse, 1980, *Psylliodes frivaldszkyi* Weise, 1888, *Psylliodes pyritosa* Kutschera, 1864, *Sclerophaedon carpathicus* (Weise, 1875), *Timarcha gibba* (Hagenbach, 1825), *Timarcha rugulosa* Herrich-Schaeffer, 1838, *Zeugophora turneri* Power, 1863.

Widely distributed species (97): *Altica lythri* Aubé, 1843, *Altica oleracea* (Linnaeus, 1758), *Batophila rubi* (Paykull, 1799), *Bromius obscurus* (Linnaeus, 1758), *Cassida flaveola* Thunberg, 1794, *Cassida hemisphaerica* Herbst, 1799, *Cassida margaritacea* Schaller, 1783, *Cassida murraea* Linnaeus, 1767, *Cassida rubiginosa* Müller, 1776, *Cassida viridis* Linnaeus, 1758, *Chaetocnema aridula* (Gyllenhal, 1827), *Chaetocnema hortensis* (Geoffroy, 1785), *Chaetocnema picipes* Stephens, 1831, *Chaetocnema sahlbergi* (Gyllenhal, 1827), *Chrysolina coerulans* (Scriba, 1791), *Chrysolina fastuosa* (Scopoli, 1763), *Chrysolina graminis* (Linnaeus, 1758), *Chrysolina haemoptera* (Linnaeus, 1758), *Chrysolina oricalcia* (Müller, 1776), *Chrysolina polita* (Linnaeus, 1758), *Chrysolina staphylaea* (Linnaeus, 1758), *Chrysomela collaris* Linnaeus, 1758, *Crepidodera aurata* (Marsham, 1802), *Crepidodera fulvicornis* (Fabricius, 1792), *Crepidodera plutus* (Latreille, 1804), *Crioceris asparagi* (Linnaeus, 1758), *Crioceris duodecimpunctata* (Linnaeus, 1758), *Cryptocephalus coryli* (Linnaeus, 1758), *Cryptocephalus decemmaculatus* (Linnaeus, 1758), *Cryptocephalus flavipes* Fabricius, 1781, *Cryptocephalus fulvus* (Goeze, 1777), *Cryptocephalus labiatus* (Linnaeus, 1761), *Cryptocephalus nitidus* (Linnaeus, 1758), *Cryptocephalus pini* (Linnaeus, 1758), *Cryptocephalus pusillus* Fabricius, 1777, *Cryptocephalus rufipes* (Goeze, 1777), *Donacia aquatica* (Linnaeus, 1758), *Donacia cinerea* Herbst, 1784, *Donacia impressa* Paykull, 1799, *Donacia thalassina* Germar, 1811, *Donacia vulgaris* Zschach, 1788, *Galeruca pomonae* (Scopoli, 1763), *Galerucella calmariensis* (Linnaeus, 1767), *Galerucella lineola* (Fabricius, 1781), *Galerucella nymphaeae* (Linnaeus, 1758), *Gastrophysa polygoni* (Linnaeus, 1758), *Gastrophysa viridula* (De Geer, 1775), *Gonioctena quinquepunctata* (Fabricius, 1787), *Hippuriphila modeeri* (Linnaeus, 1761), *Lema cyanella* (Linnaeus, 1758), *Leptinotarsa decemlineata* (Say, 1824), *Lilioceris lilii* (Scopoli, 1763), *Lochmaea crataegi* (Forster, 1771), *Lochmaea suturalis* (Thomson, 1866), *Longitarsus anchusae* (Paykull 1799), *Longitarsus atricillus* (Linnaeus, 1761), *Longitarsus brunneus* (Duftschmid, 1825), *Longitarsus dorsalis* (Fabricius, 1781), *Longitarsus exoletus* (Linnaeus, 1758), *Longitarsus ferrugineus* (Foudras, 1869), *Longitarsus jacobaeae* Waterhouse, 1858, *Longitarsus luridus* (Scopoli, 1763), *Longitarsus lycopi* (Foudras, 1860), *Longitarsus nasturtii* (Fabricius, 1792), *Longitarsus pratensis* (Panzer, 1794), *Longitarsus quadriguttatus* (Pontoppidan, 1765), *Longitarsus succineus* (Foudras, 1860), *Longitarsus tabidus* (Fabricius, 1775), *Luperus longicornis* (Fabricius, 1761), *Lythraria salicariae* (Paykull, 1800), *Neocrepidodera ferruginea* (Scopoli, 1763), *Neocrepidodera transversa* (Marsham, 1802), *Oulema melanopus* (Linnaeus, 1758), *Phaedon armoraciae* (Linnaeus, 1758), *Phaedon cochleariae* (Fabricius, 1792), *Phratora laticollis* (Suffrian, 1851), *Phratora vitellinae* (Linnaeus, 1758), *Phyllotreta exclamationis* (Thunberg, 1784), *Phyllotreta nemorum* (Linnaeus, 1758), *Phyllotreta tetrastigma* (Comolli, 1837), *Phyllotreta undulata* Kutschera, 1860, *Phyllotreta vittula* (Redtenbacher, 1849), *Plagiosterna aenea* (Linnaeus, 1758), *Plateumaris affinis* (Kunze, 1818), *Plateumaris consimilis* (Schrank, 1781), *Plateumaris sericea* (Linnaeus, 1761), *Prasocuris phellandrii* (Linnaeus, 1758), *Psylliodes affinis* (Paykull, 1799), *Psylliodes chrysocephalus* (Linnaeus, 1758), *Psylliodes napi* (Fabricius, 1792), *Psylliodes picina* (Marsham, 1802), *Pyrrhalta viburni* (Paykull, 1799), *Sermylassa halensis* (Linnaeus, 1767), *Sphaeroderma testaceum* (Fabricius, 1775), *Zeugophora flavicollis* (Marsham, 1802), *Zeugophora scutellaris* Suffrian, 1840, *Zeugophora subspinosa* (Fabricius, 1781).

Southern distribution (103): *Altica helianthemi* (Allard, 1859), *Altica tamaricis* Schrank, 1785, *Aphthona abdominalis* (Duftschmid, 1825), *Aphthona atrovirens* (Förster, 1849), *Aphthona cyparissiae* (Koch, 1803), *Aphthona herbrigada* (Curtis, 1837), *Aphthona pallida* (Bach, 1856), *Aphthona pygmaea* (Kutschera, 1861), *Aphthona venustula* (Kutschera, 1861), *Apteropeda orbiculata* (Marsham, 1802), *Calomicrus circumfusus* (Marsham, 1802), *Calomicrus pinicola* (Duftschmid, 1825), *Cassida panzeri* Weise, 1907, *Chaetocnema arida* Foudras, 1860, *Chaetocnema obesa* (Boieldieu, 1859), *Chaetocnema semicoerulea* (Koch, 1803), *Chrysolina cuprina* (Duftschmid, 1825), *Chrysolina hemisphaerica* (Germar, 1817), *Chrysolina herbacea* (Duftschmid, 1825), *Chrysolina hyperici* (Forster, 1771), *Chrysolina marginata* (Linnaeus, 1758), *Chrysolina rufa* (Duftschmid, 1825), *Chrysomela cuprea* Fabricius, 1775, *Chrysomela saliceti* (Weise, 1884), *Chrysomela vigintipunctata* (Scopoli, 1763), *Coptocephala rubicunda* (Laicharting, 1781), *Crepidodera aurea* (Geoffroy, 1785), *Crepidodera lamina* (Bedel, 1901), *Crepidodera nitidula* (Linnaeus, 1758), *Cryptocephalus biguttatus* (Scopoli, 1763), *Cryptocephalus frontalis* Marsham, 1802, *Cryptocephalus laetus* Fabricius, 1792, *Cryptocephalus primarius* Harold, 1872, *Cryptocephalus pygmaeus* Fabricius, 1792, *Cryptocephalus querceti* Suffrian, 1848, *Cryptocephalus quinquepunctatus* (Scopoli, 1763), *Cryptocephalus saliceti* Zebe, 1855, *Cryptocephalus schaefferi* Schrank, 1789, *Cryptocephalus sexpunctatus* (Linnaeus, 1758), *Cryptocephalus signatifrons* Suffrian, 1847, *Cryptocephalus variegatus* Fabricius, 1781, *Cryptocephalus vittatus* Fabricius, 1775, *Derocrepis rufipes* (Linnaeus, 1758), *Dibolia foersteri* Bach, 1859, *Donacia springeri* Müller, 1916, *Epitrix atropae* Foudras, 1860, *Epitrix intermedia* Foudras, 1860, *Galeruca laticollis* (Sahlberg, 1837), *Galerucella tenella* (Linnaeus, 1761), *Gonioctena intermedia* (Helliesen, 1913), *Gonioctena linnaeana* (Schrank, 1781), *Gonioctena pallida* (Linnaeus, 1758), *Gonioctena viminalis* (Linnaeus, 1758), *Hermaeophaga mercurialis* (Fabricius, 1792), *Hispa atra* Linnaeus, 1767, *Hydrothassa glabra* (Herbst, 1783), *Labidostomis humeralis* (Schneider, 1792), *Labidostomis lucida* (Germar, 1823), *Labidostomis pallidipennis* (Gebler, 1839), *Labidostomis tridentata* (Linnaeus, 1758), *Lachnaia sexpunctata* (Scopoli, 1763), *Lilioceris merdigera* (Linnaeus, 1758), *Longitarsus absynthii* Kutschera, 1862, *Lachnaia echii* (Koch, 1803), *Lachnaia lateripunctatus* (Rosenhauer, 1856), *Lachnaia longiseta* Weise, 1889, *Lachnaia membranaceus* (Foudras, 1860), *Lachnaia minusculus* (Foudras, 1860), *Lachnaia nanus* (Foudras, 1860), *Lachnaia pellucidus* (Foudras, 1860), *Lachnaia pulmonariae* Weise, 1893, *Lachnaia scutellaris* (Rey, 1873), *Luperus flavipes* (Linnaeus, 1767), *Mantura mathewsi* (Curtis, 1834), *Neocrepidodera femorata* (Gyllenhal, 1813), *Ochrosis ventralis* (Illiger, 1807), *Oomorphus concolor* (Sturm, 1807), *Orsodacne cerasi* (Linnaeus, 1758), *Pachnephorus pilosus* (Rossi, 1790), *Pachybrachis hieroglyphicus* (Laicharting, 1781), *Pachybrachis hippophaes* Suffrian, 1848, *Pachybrachis picus* Weise, 1882, *Pachybrachis sinuatus* Mulsant, 1859, *Pachybrachis tesselatus* (Olivier, 1791), *Phaedon laevigatus* (Duftschmid, 1825), *Phratora tibialis* (Suffrian, 1851), *Phratora vulgatissima* (Linnaeus, 1758), *Phyllotreta christinae* Heikertinger, 1941, *Phyllotreta ochripes* (Curtis, 1837), *Phyllotreta procera* (Redtenbacher, 1849), *Phyllotreta punctulata* (Marsham, 1802), *Plagiodera versicolora* (Laicharting, 1781), *Psylliodes chalcomera* (Illiger, 1807), *Psylliodes instabilis* Foudras, 1860, *Psylliodes isatidis* Heikertinger, 1912, *Psylliodes thlaspis* Foudras, 1860, *Smaragdina affinis* (Illiger, 1794), *Smaragdina flavicollis* (Charpentier, 1825), *Sphaeroderma rubidum* (Graëlls, 1858), *Timarcha goettingensis* (Linnaeus, 1758), *Timarcha metallica* (Laicharting, 1781), *Timarcha pratensis* (Duftschmid, 1825), *Zeugophora frontalis* Suffrian, 1840.

South-Eastern (52): *Bruchidius marginalis* (Fabricius, 1776), *Bruchus atomarius* (Linnaeus, 1761), *Cassida ferruginea* Goeze, 1777, *Cassida rufovirens* Suffrian, 1844, *Cassida sanguinolenta* Müller, 1776, *Cassida subferruginea* (Schrank, 1776), *Cassida subreticulata* Suffrian, 1844, *Cassida vibex* Linnaeus, 1767, *Chrysochus asclepiadeus* (Pallas, 1773), *Chrysolina geminata* (Paykull, 1799), *Chrysolina kuesteri* (Helliesen, 1912), *Chrysolina lichenis* (Richter, 1820), *Chrysolina sturmi* (Westhoff, 1882), *Chrysolina varians* (Schaller, 1783), *Chrysomela populi* Linnaeus, 1758, *Chrysomela tremulae* Fabricius, 1783, *Clytra laeviuscula* Ratzeburg, 1837, *Clytra quadripunctata* (Linnaeus, 1758), *Coptocephala unifasciata* (Scopoli, 1763), *Cryptocephalus aureolus* Suffrian, 1847, *Cryptocephalus bilineatus* (Linnaeus, 1767), *Cryptocephalus chrysopus* Gmelin, 1788, *Cryptocephalus cordiger* (Linnaeus, 1758), *Cryptocephalus elegantulus* Gravenhorst, 1807, *Cryptocephalus exiguus* Schneider, 1792, *Cryptocephalus frenatus* Laicharting, 1781, *Cryptocephalus hypochaeridis* (Linnaeus, 1758), *Cryptocephalus marginatus* Fabricius, 1781, *Cryptocephalus moraei* (Linnaeus, 1758), *Cryptocephalus octopunctatus* (Scopoli, 1763), *Cryptocephalus violaceus* Laicharting, 1781, *Cryptocephalus vittula* Suffrian, 1848, *Dibolia depressiuscula* Letzner, 1847, *Dibolia femoralis* Redtenbacher, 1849, *Dibolia rugulosa* Redtenbacher, 1849, *Galeruca tanaceti* (Linnaeus, 1758), *Labidostomis longimana* (Linnaeus, 1761), *Longitarsus apicalis* (Beck, 1817), *Longitarsus ballotae* (Marsham, 1802), *Longitarsus foudrasi* Weise, 1893, *Longitarsus melanocephalus* (De Geer, 1775), *Longitarsus nigrofasciatus* (Goeze, 1777), *Longitarsus obliteratus* (Rosenhauer, 1847), *Longitarsus salviae* Gruev, 1975, *Mantura obtusata* (Gyllenhal, 1813), *Minota obesa* (Waltl, 1839), *Oulema gallaeciana* (Heyden, 1870), *Phyllotreta diademata* Foudras, 1860, *Phyllotreta nodicornis* (Marsham, 1802), *Podagrica fuscicornis* (Linnaeus, 1767), *Smaragdina aurita* (Linnaeus, 1767), *Smaragdina salicina* (Scopoli, 1763).

South-Western (12): *Apteropeda globosa* (Illiger, 1794), *Apteropeda splendida* Allard, 1860, *Bruchus rufipes* Herbst, 1783, *Cryptocephalus ocellatus* Drapiez, 1819, *Dibolia cryptocephala* (Koch, 1803), *Donacia bicolora* Zschach, 1788, *Donacia simplex* Fabricius, 1775, *Longitarsus aeruginosus* (Foudras, 1860), *Longitarsus ganglbaueri* Heikertinger, 1912, *Longitarsus rubiginosus* (Foudras, 1860), *Mniophila muscorum* (Koch, 1803), *Timarcha tenebricosa* (Fabricius, 1775).

Northern (8): *Galerucella grisescens* (Joannis, 1865), *Hydrothassa hannoverana* (Fabricius, 1775), *Longitarsus plantagomaritimus* Dollman, 1912, *Mantura chrysanthemi* (Koch, 1803), *Phaedon concinnus* Stephens, 1831, *Phyllotreta armoraciae* (Koch, 1803), *Psylliodes crambicola* Lohse, 1954, *Psylliodes marcida* (Illiger, 1807).

Eastern (16): *Aphthona nigriscutis* Foudras, 1860, *Aphthona placida* (Kutschera, 1854), *Cassida berolinensis* Suffrian, 1844, *Chrysolina analis* (Linnaeus, 1767), *Chrysolina marcasitica* (Germar, 1824), *Chrysolina umbratilis* (Weise, 1887), *Colaphus sophiae* (Schaller, 1783), *Crioceris quatuordecimpunctata* (Scopoli, 1763), *Cryptocephalus distinguendus* Schneider, 1792, *Cryptocephalus quadripunctatus* Olivier, 1808, *Cryptocephalus virens* Suffrian, 1847, *Dibolia schillingi* (Letzner, 1847), *Galeruca dahli* (Joannis, 1865), *Luperus saxonicus* (Gmelin, 1790), *Phyllotreta scheuchi* Heikertinger, 1941, *Psylliodes hyoscyami* (Linnaeus, 1758).

South-East quarter (52): *Altica carduorum* Guérin-Meneville, 1858, *Aphthona flava* Guillebeau, 1895, *Aphthona franzi* Heikertinger, 1944, *Aphthona lacertosa* (Rosenhauer, 1847), *Aphthona semicyanea* Allard, 1859, *Aphthona stussineri* Weise, 1888, *Argopus ahrensi* (Germar, 1817), *Cassida atrata* Fabricius, 1787, *Cassida inquinata* Brullé, 1832, *Chaetocnema arenacea* (Allard, 1860), *Chaetocnema chlorophana* (Duftschmid, 1825), *Chaetocnema conducta* (Motschulsky, 1838), *Chaetocnema major* (Jacquelin-Duval, 1852), *Chrysolina carpathica* (Fuss, 1856), *Chrysolina chalcites* (Germar, 1824), *Chrysolina globosa* (Panzer, 1805), *Chrysolina olivieri* (Bedel, 1892), *Chrysolina rossia* (Illiger, 1802), *Chrysolina rufoaenea* (Suffrian, 1851), *Coptocephala chalybaea* (Germar, 1824), *Coptocephala scopolina* (Linnaeus, 1767), *Crioceris quinquepunctata* (Scopoli, 1763), *Cryptocephalus apicalis* Gebler, 1830, *Cryptocephalus connexus* Olivier, 1807, *Cryptocephalus imperialis* Laicharting, 1781, *Cryptocephalus laevicollis* Gebler, 1830, *Cryptocephalus quatuordecimmaculatus* Schneider, 1792, *Entomoscelis adonidis* (Pallas, 1771), *Entomoscelis sacra* (Linnaeus, 1758), *Exosoma lusitanica* (Linnaeus, 1767), *Galeruca rufa* Germar, 1824, *Gonioctena fornicata* (Brüggemann, 1873), *Gonioctena gobanzi* (Reitter, 1902), *Labidostomis cyanicornis* (Germar, 1817), *Lachnaia italica* (Weise, 1882), *Longitarsus cerinthes* (Schrank, 1798), *Longitarsus languidus* Kutschera, 1863, *Longitarsus linnaei* (Duftschmid, 1825), *Longitarsus medvedevi* Shapiro, 1956, *Longitarsus minimus* Kutschera, 1864, *Longitarsus monticola* Kutschera, 1863, *Longitarsus pallidicornis* Kutschera, 1863, *Neocrepidodera brevicollis* (J. Daniel, 1904), *Pachnephorus tesselatus* (Duftschmid, 1825), *Pachnephorus villosus* (Duftschmid, 1825), *Phyllobrotica adusta* (Creutzer, 1799), *Phyllotreta ganglbaueri* Heikertinger, 1909, *Podagrica menetriesi* (Faldermann, 1837), *Psylliodes attenuata* (Koch, 1803), *Psylliodes brisouti* Bedel, 1898, *Psylliodes gibbosa* Allard, 1860, *Tituboea macropus* (Illiger, 1800).

South-West quarter (5): *Bruchidius varius* (Olivier, 1795), *Batophila aerata* (Marsham, 1802), *Longitarsus brisouti* Heikertinger, 1912, *Mantura horioni* Heikertinger, 1940, and *Podagrica fuscipes* (Fabricius, 1775).

Fragmented (37): *Agelastica alni* (Linnaeus, 1758), *Altica aenescens* (Weise, 1888), *Altica palustris* (Weise, 1888), *Altica quercetorum* Foudras, 1860, *Aphthona nonstriata* (Goeze, 1777), *Bruchus loti* Paykull, 1800, *Cassida denticollis* Suffrian, 1844, *Cassida nebulosa* Linnaeus, 1758, *Cassida nobilis* Linnaeus, 1758, *Cassida prasina* Illiger, 1798, *Cassida sanguinosa* Suffrian, 1844, *Cassida stigmatica* Suffrian, 1844, *Cassida vittata* Villiers, 1789, *Chaetocnema concinna* (Marsham, 1802), *Chaetocnema mannheimeri* (Gyllenhal, 1827), *Cheilotoma musciformis* (Goeze, 1777), *Chrysolina sanguinolenta* (Linnaeus, 1758), *Cryptocephalus bipunctatus* (Linnaeus, 1758), *Cryptocephalus janthinus* Germar, 1824, *Cryptocephalus parvulus* Müller, 1776, *Dibolia occultans* (Koch, 1803), *Donacia clavipes* Fabricius, 1793, *Donacia crassipes* Fabricius, 1775, *Donacia marginata* Hoppe, 1795, *Donacia sparganii* Ahrens, 1810, *Epitrix pubescens* (Koch, 1803), *Galerucella aquatica* (Fourcroy, 1785), *Galerucella pusilla* (Duftschmid, 1825), *Longitarsus holsaticus* (Linnaeus, 1758), *Longitarsus kutscherae* (Rye, 1872), *Phyllotretaq atra* (Fabricius, 1775), *Phyllotretaq cruciferae* (Goeze, 1777), *Phyllotretaq ochripes* (Curtis, 1837), *Phyllotretaq striolata* (Fabricius, 1803), *Plateumaris rustica* (Kunze, 1818), *Psylliodes cuprea* (Koch, 1803), *Psylliodes dulcamarae* (Koch, 1803).

Montane (18): *Aphthona ovata* Foudras, 1860, *Calomicrus gularis* (Gredler, 1857), *Chaetocnema angustula* (Rosenhauer, 1847), *Chrysolina aurichalcea* (Mannerheim, 1825), *Cryptocephalus nitidulus* Fabricius, 1787, *Longitarsus helvolus* Kutschera, 1863, *Luperus viridipennis* Germar, 1824, *Luperus xanthopoda* (Schrank, 1781), *Oreina alpestris* (Schummel, 1844), *Oreina bifrons* (Fabricius, 1792), *Oreina cacaliae* (Schrank, 1785), *Oreina intricata* (Germar, 1824), *Oreina speciosa* (Linnaeus, 1767), *Oreina speciosissima* (Scopoli, 1763), *Psylliodes glabra* (Duftschmid, 1825), *Psylliodes toelgi* Heikertinger, 1914, *Psylliodes vindobonensis* Heikertinger, 1914, *Sclerophaedon carniolicus* (Germar, 1824).

Alpine (28): *Chrysolina globosa* (Panzer, 1805), *Chrysolina latecincta* (Demaison, 1896), *Chrysolina relucens* (Rosenhauer, 1847), *Cryptocephalus albolineatus* Suffrian, 1847, *Cryptocephalus strigosus* Germar, 1823, *Gonioctena holdhausi* (Leeder, 1950), *Lilioceris tibialis* (Villa, 1838), *Longitarsus rubellus* (Foudras, 1860), *Neocrepidodera cyanescens* (Duftschmid, 1825), *Neocrepidodera cyanipennis* (Kutschera, 1860), *Neocrepidodera melanostoma* (Redtenbacher, 1849), *Neocrepidodera norica* (Weise, 1860), *Neocrepidodera obirensis* (Ganglbauer, 1897), *Neocrepidodera periolerii* (Kutschera, 1860), *Neocrepidodera rhaetica* (Kutschera, 1860), *Oreina frigida* (Weise, 1883), *Oreina gloriosa* (Fabricius, 1781), *Oreina liturata* (Scopoli, 1763), *Oreina melanocephala* (Duftschmid, 1825), *Oreina virgulata* (Germar, 1824), *Oreina viridis* (Duftschmid, 1825), *Oreina vittigera* (Suffrian, 1851), *Orestia alpina* (Germar, 1824), *Phaedon segnis* Wesie, 1884, *Psylliodes aerea* Foudras, 1860, *Psylliodes picipes* Redtenbacher, 1849, *Psylliodes rambouseki* Heikertinger, 1909, *Psylliodes subaenea* Kutschera, 1864.

Scattered (53): *Acanthoscelides obtectus* (Say, 1831), *Altica brevicollis* Foudras, 1860, *Aphthona atrocaerulea* (Stephens, 1831), *Aphthona euphorbiae* (Schrank, 1781), *Aphthona lutescens* (Gyllenhal, 1813), *Aphthona violacea* (Koch, 1803), *Bruchidius cisti* (Fabricius, 1775), *Bruchidius villosus* (Fabricius, 1792), *Bruchus affinis* Frölich, 1799, *Bruchidius brachialis* Fahraeus, 1839, *Bruchidius luteicornis* Illiger, 1794, *Bruchidius rufimanus* Boheman, 1833, *Cassida azurea* Fabricius, 1801, *Cassida canaliculata* Laicharting, 1781, *Cassida fastuosa* Schaller, 1783, *Cassida pannonica* Suffrian, 1844, *Chaetocnema aerosa* (Letzner, 1846), *Chaetocnema confusa* (Boheman, 1851), *Chrysolina brunsvicensis* (Gravenhorst, 1807), *Chaetocnema cerealis* (Linnaeus, 1767), *Cryptocephalus caerulaescens* Sahlberg, 1839, *Cryptocephalus ochroleucus* Stephens, 1834, *Cryptocephalus pallifrons* Gyllenhal, 1813, *Cryptocephalus populi* Suffrian, 1848, *Cryptocephalus punctiger* Paykull, 1799, *Cryptocephalus quadripustulatus* Gyllenhal, 1813, *Dibolia cyanoglossi* (Koch, 1803), *Donacia antiqua* Kunze, 1818, *Donacia brevicornis* Ahrens, 1810, *Donacia dentata* Hoppe, 1795, *Donacia malinowskyi* Ahrens, 1810, *Donacia tomentosa* Ahrens, 1810, *Donacia versicolorea* (Brahm, 1790), *Longitarsus agilis* (Rye, 1868), *Longitarsus curtus* (Allard, 1860), *Longitarsus fulgens* (Foudras, 1860), *Longitarsus gracilis* Kutschera, 1864, *Longitarsus niger* (Koch, 1803), *Longitarsus nigerrimus* (Gyllenhal, 1827), *Longitarsus ochroleucus* (Marsham, 1802), *Longitarsus reichei* (Allard, 1860), *Longitarsus symphyti* Heikertinger, 1912, *Macroplea appendiculata* (Panzer, 1794), *Mantura rustica* (Linnaeus, 1767), *Neocrepidodera motschulskii* Konstantinov, 1991, *Neocrepidodera nigritula* (Gyllenhal, 1813), *Oulema erichsonii* (Suffrian, 1841), *Phratora atrovirens* (Cornelius, 1857), *Phyllotreta dilatata* Thomson, 1866, *Phyllotreta flexuosa* (Illiger, 1794), *Psylliodes laticollis* Kutschera, 1864, *Psylliodes luteola* (Müller, 1776), *Xanthogaleruca luteola* (Müller, 1776).

Unusual (50): *Altica carinthiaca* (Weise, 1888), *Altica impressicollis* (Reiche, 1862), *Chaetocnema compressa* (Letzner, 1847), *Chaetocnema procerula* (Rosenhauer, 1856), *Chaetocnema tibialis* (Illiger, 1807), *Chrysolina carnifex* (Fabricius, 1792), *Chrysolina fuliginosa* (Olivier, 1807), *Chrysolina gypsophilae* (Küster, 1845), *Chrysolina limbata* (Fabricius, 1775), *Chrysomela lapponica* (Linnaeus, 1758), *Cryptocephalus cyanipes* Suffrian, 1847, *Cryptocephalus elongatus* Germar, 1824, *Cryptocephalus sericeus* (Linnaeus, 1758), *Dibolia timida* (Illiger, 1807), *Donacia obscura* Gyllenhal, 1813, *Donacia semicuprea* Panzer, 1796, *Galeruca interrupta* Illiger, 1802, *Galeruca melanocephala* Ponza, 1805, *Gonioctena decemnotata* (Marsham, 1802), *Gonioctena interposita* (Franz & Palmén, 1950), *Gonioctena olivacea* (Forster, 1771), *Lochmaea caprea* (Linnaeus, 1758), *Longitarsus australis* (Mulsant & Rey, 1802), *Longitarsus lewisii* (Baly, 1874), *Longitarsus noricus* Leonardi, 1976, *Longitarsus parvulus* (Paykull, 1799), *Longitarsus suturellus* (Duftschmid, 1825), *Luperus luperus* (Sulzer, 1776), *Macroplea mutica* (Fabricius, 1792), *Orsodacne lineola* (Panzer, 1896), *Oulema duftschmidi* (Redtenbacher, 1874), *Oulema rufocyanea* (Suffrian, 1847), *Oulema tristis* (Herbst, 1786), *Pachybrachis fimbriolatus* Suffrian, 1848, *Phaedon pyritosus* (Rossi, 1792), *Phyllotreta astrachanica* Lopatin, 1977, *Phyllotreta austriaca* Heikertinger, 1909, *Plateumaris bracata* (Scopoli, 1772), *Plateumaris discolor* (Panzer, 1795), *Podagrica malvae* (Illiger, 1807), *Prasocuris junci* (Brahm, 1790), *Prasocuris marginella* (Linnaeus, 1758), *Psylliodes cucullata* (Illiger, 1807), *Psylliodes cupreata* (Duftschmid, 1825), *Psylliodes reitteri* Weise, 1888, *Psylliodes sophiae* Heikertinger, 1914, *Smaragdina diversipes* Letzner, 1839, *Smaragdina xanthaspis* (Germar, 1824), *Spermophagus calystegiae* (Lukjanov & Ter-Minassian, 1957), *Spermophagus sericeus* (Fourcroy, 1785).
